# Brain Renin-Angiotensin System and Microglial Polarization: Implications for Aging and Neurodegeneration

**DOI:** 10.3389/fnagi.2017.00129

**Published:** 2017-05-03

**Authors:** Jose L. Labandeira-Garcia, Ana I. Rodríguez-Perez, Pablo Garrido-Gil, Jannette Rodriguez-Pallares, Jose L. Lanciego, Maria J. Guerra

**Affiliations:** ^1^Laboratory of Neuroanatomy and Experimental Neurology, Department of Morphological Sciences, Center for Research in Molecular Medicine and Chronic Diseases (CIMUS), University of Santiago de CompostelaSantiago de Compostela, Spain; ^2^Networking Research Center on Neurodegenerative Diseases (CIBERNED)Madrid, Spain; ^3^Neurosciences Division, Center for Applied Medical Research (CIMA), University of NavarraPamplona, Spain

**Keywords:** angiotensin, microglia, NADPH-oxidase, neuroinflammation, neuroprotection, Nox, oxidative stress, Parkinson

## Abstract

Microglia can transform into proinflammatory/classically activated (M1) or anti-inflammatory/alternatively activated (M2) phenotypes following environmental signals related to physiological conditions or brain lesions. An adequate transition from the M1 (proinflammatory) to M2 (immunoregulatory) phenotype is necessary to counteract brain damage. Several factors involved in microglial polarization have already been identified. However, the effects of the brain renin-angiotensin system (RAS) on microglial polarization are less known. It is well known that there is a “classical” circulating RAS; however, a second RAS (local or tissue RAS) has been observed in many tissues, including brain. The locally formed angiotensin is involved in local pathological changes of these tissues and modulates immune cells, which are equipped with all the components of the RAS. There are also recent data showing that brain RAS plays a major role in microglial polarization. Level of microglial NADPH-oxidase (Nox) activation is a major regulator of the shift between M1/proinflammatory and M2/immunoregulatory microglial phenotypes so that Nox activation promotes the proinflammatory and inhibits the immunoregulatory phenotype. Angiotensin II (Ang II), via its type 1 receptor (AT1), is a major activator of the NADPH-oxidase complex, leading to pro-oxidative and pro-inflammatory effects. However, these effects are counteracted by a RAS opposite arm constituted by Angiotensin II/AT2 receptor signaling and Angiotensin 1–7/Mas receptor (MasR) signaling. In addition, activation of prorenin-renin receptors may contribute to activation of the proinflammatory phenotype. Aged brains showed upregulation of AT1 and downregulation of AT2 receptor expression, which may contribute to a pro-oxidative pro-inflammatory state and the increase in neuron vulnerability. Several recent studies have shown interactions between the brain RAS and different factors involved in microglial polarization, such as estrogens, Rho kinase (ROCK), insulin-like growth factor-1 (IGF-1), tumor necrosis factor α (TNF)-α, iron, peroxisome proliferator-activated receptor gamma, and toll-like receptors (TLRs). Metabolic reprogramming has recently been involved in the regulation of the neuroinflammatory response. Interestingly, we have recently observed a mitochondrial RAS, which is altered in aged brains. In conclusion, dysregulation of brain RAS plays a major role in aging-related changes and neurodegeneration by exacerbation of oxidative stress (OS) and neuroinflammation, which may be attenuated by pharmacological manipulation of RAS components.

## Introduction

In the late 19th century, Franz Nissl first described microglia as staebchenzellen (rod cells) for their rod-shaped nuclei. In 1913, Santiago Ramón y Cajal described these cells as a “third element”, and in 1919 microglial cells were characterized by his disciple Pío del Río Hortega as a phagocytic and distinct non-neural and non-astrocytic population. During embryonic development, primitive yolk sac myeloid progenitors enter the brain and differentiate into microglial cells (Alliot et al., 1999; Ginhoux et al., 2010). It is usually estimated that around 10% of the adult brain cells are microglial cells (Carson et al., 2006; Kettenmann et al., 2011). In the adult mouse brain, microglia constitute 5%–12% of all glial cells, although these cells are not evenly distributed throughout the brain, ranging from 5% in the cerebral cortex to 12% in the substantia nigra (Lawson et al., 1990; Mittelbronn et al., 2001; Harry and Kraft, 2012). There are tissue-specific macrophages in practically all tissues of the body (Gautier et al., 2012), and microglial cells have been considered as the resident macrophages in the brain. Consistent with this, the resident microglia mediate the brain immune and inflammatory responses (Hu et al., 2014; Prinz and Priller, 2014). Although microglia and brain macrophages express several common protein markers, microglial cells have a particular molecular signature, which is different to other circulating and tissue-resident macrophagic cells (Hickman et al., 2013; Butovsky et al., 2014).

Microglial cells have been classically described to exist in two states, resting and activated state. In the healthy brain, neurons produce several immunosuppressive proteins, which keep microglia in a classical inactivated state (Harrison et al., 1998; Hoek et al., 2000). However, the initial concept of resting microglia has been modified, and recent studies have shown that the classical “resting” microglia are highly active by scanning of the surrounding region for disturbances in physiological conditions (Davalos et al., 2005; Nimmerjahn et al., 2005). Furthermore, it is now generally admitted that the term activation includes a set of several “activated” states. The classical concept of either “good” or “bad” activation states of microglia has to be reconsidered, and substituted by a range of different types of functional activation. Microglia can develop a number of different phenotypes and functions to preserve brain homeostasis depending on their environment.

## Microglial Polarization

The dual role of microglia is associated with different polarization of microglia under different context, particularly after brain injury (Hu et al., 2014; Franco and Fernández-Suárez, 2015; Kim et al., 2015). Microglia can develop into proinflammatory/classically activated (M1) or anti-inflammatory/alternatively activated (M2) phenotypes depending on the signals present at different stages after brain lesions. M1/proinflammatory microglia produces proinflammatory mediators and free radicals that exacerbate neuronal death. Alternatively, M2/immunoregulatory microglia induce brain repair and regeneration, produce growth factors and anti-inflammatory cytokines to protect neurons and resolve inflammation. In addition, as primarily described in the periphery, several subclasses of M2/immunoregulatory activation have been identified. The M2a activation state has a main function of suppression of inflammation. A second state of alternative activation is classified as “M2c”, which has been suggested to restore the tissue after the inflammatory process has been attenuated (Gordon, 2003; Colton, 2009; Sica and Mantovani, 2012). The class termed “M2b” is least understood; M2b has been involved in both pro- or anti-inflammatory responses and related to memory immune responses (Mantovani et al., 2004; Edwards et al., 2006). The functional polarization of microglia is modulated by several receptors, transcription factors, acute phase proteins, and metabolic states. Toll-like receptors (TLRs), nucleotide-binding oligomerization domains (NODs) and NOD-like receptors are known to play a major role in microglia-mediated inflammation (Ransohoff and Perry, 2009; Ransohoff and Brown, 2012). Upregulation and release of reactive oxygen species (ROS) and activation of inducible nitric oxide synthase (iNOS), followed by the release of reactive nitrogen species (RNS), are hallmarks of M1 macrophages/ microglia (MacMicking et al., 1997). Upregulation of the enzyme arginase 1 (Arg1) is considered as an specific marker of M2 macrophages/microglia (Sica and Bronte, 2007; Chhor et al., 2013).

An adequate progression from the proinflammatory/M1 to immunoregulatory/M2 phenotype is necessary to efficiently counteract brain lesions. However, when this process is dysregulated, the persistent release of inflammatory cytokines and ROS induces neuron death and enhances brain damage (Kigerl et al., 2009). Interestingly, microglial activation and enhanced neuroinflammatory responses have been observed in major neurodegenerative diseases (Frank-Cannon et al., 2009). Persistently proinflammatory polarized microglia and release of proinflammatory cytokines leads to a pro-oxidative and proinflammatory milieu and enhances neurodegeneration (Heneka et al., 2015; Tang and Le, 2016). New therapeutical strategies are necessary to modulate microglial activation and drive microglia polarization to a protective phenotype (Koenigsknecht-Talboo and Landreth, 2005; Porrini et al., 2015).

Proinflammatory phenotype polarization can be induced by stimulation with compounds such as the TLR-4 agonist lipopolysaccharide (LPS), interferon (IFN)-γ, Interleukin (IL)-17A and tumor necrosis factor-α (TNF)-α. Alternatively, stimuli such as IL-4, IL-10, or transforming growth factor β (TGF)-β induce the M2/immunoregulatory phenotype. However, the effects of the brain renin-angiotensin system (RAS) on microglial polarization are less known. It has been shown that angiotensin modulates immune cells in peripheral tissues, and that inflammatory cells have all RAS components, including angiotensin II (Ang II). Ang II receptor antagonists are modulators of macrophagic polarization in different tissues such adipose tissue, lung, kidney, or tumors (Fujisaka et al., 2011; Ma et al., 2011; Liu et al., 2012; Shrestha et al., 2016). There are also recent data showing that brain RAS plays a major role in microglial polarization.

## The Renin-Angiotensin System

**Figure 1:**
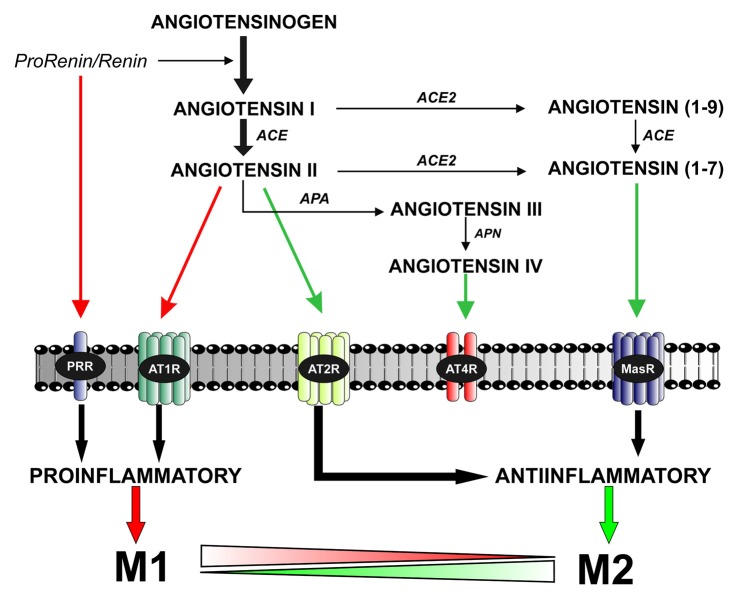
**The brain renin–angiotensin system (RAS).** Angiotensin II is formed by the sequential action of two enzymes prorenin-renin (that forms angiotensin I) and ACE on the precursor protein angiotensinogen. Renin and its precursor prorenin also act on specific PRR receptors. In addition to angiotensin II, several angiotensin peptides such as Angiotensin (1–7), angiotensin III and angiotensin IV are formed from angiotensin I and II by the action of ACE2, APA and APN. Angiotensin II, via AT1 receptors, induces pro-inflammatory effects and M1 phenotype. However, these effects are counteracted by a RAS opposite arm constituted by angiotensin II/AT2 receptor signaling and Angiotensin 1–7/MasR signaling. In addition, activation of prorenin-renin receptors may contribute to activation of the proinflammatory phenotype. Abbreviations: ACE, angiotensin converting enzyme; ACE2, angiotensin converting enzyme 2; APA, aminopeptidase A; APN, aminopeptidase N; AT1R, angiotensin type 1 receptor; AT2R, angiotensin type 2 receptor; AT4R, angiotensin type 4 receptor; MasR, Mas receptor; PRR, prorenin/renin receptor.

The RAS was initially considered as a circulating humoral system, with functions in regulating blood pressure and in sodium and water homeostasis. Ang II, which is the most important effector peptide of the RAS, is formed by the sequential action of two enzymes (renin and angiotensin converting enzyme, ACE) on angiotensinogen. Ang II acts via type 1 and 2 (AT1 and AT2) receptors (Unger et al., 1996; Oro et al., 2007; Jones et al., 2008). AT1 and AT2 receptors have usually opposite effects on several cell functions (Chabrashvili et al., 2003; Jones et al., 2008). A number of data suggest, however, that interactions between AT1 and AT2 receptors may be more complex, and additional studies are necessary. Furthermore, new RAS components have emerged, which appear to contribute to further regulate the system (see below; Figure 1).

In addition to the “classical” circulating RAS, a second RAS (local or tissue RAS) has been observed in several tissues. A local independent RAS has also been observed in the brain (Ganong, 1994; Re, 2004). The local systems have all components observed in the circulating RAS. The local Ang II plays a relevant function in pathological changes of the corresponding tissues (Ruiz-Ortega et al., 2001; Suzuki et al., 2003). Tissue Ang II, via AT1 receptors, induces oxidative stress (OS) damage by activation of the NADPH-oxidase complex (Nox; Touyz, 2004; Garrido and Griendling, 2009). In different types of cells, PKC mediates initiation of AT1-induced Nox activation (Plumb et al., 2005; Herrera et al., 2010), which was also confirmed in microglial cells (Joglar et al., 2009). However, this initial generation of superoxide/H_2_O_2_ activates multiple downstream signals and transcription factors that may further amplify Nox oxidase activity in a multistep process that remains to be totally clarified (Seshiah et al., 2002; Balakumar and Jagadeesh, 2014). Consistent with this, we have recently shown that Nox-derived superoxide activates NFκB and the RhoA/Rho kinase pathway in microglial cells, which further increased Nox activation via p38 MAPK (Borrajo et al., 2014a; Rodriguez-Perez et al., 2015a).

More recent studies on local RAS have identified new components and mechanisms controlling RAS effects (Figure 1). New ACE homologs such as ACE2 and Chymase have been observed in different cells and tissues (Bacani and Frishman, 2006; Hamming et al., 2007). In addition to Ang II, several angiotensin peptides such as Ang (1–7), Ang III and Ang IV have been shown to have functional effects. Ang IV may act via specific AT4 receptors (Albiston et al., 2001). Ang (1–7), via a G-protein coupled receptor Mas (Santos et al., 2003), may counteract the effects of activation of AT1 by Ang II (Clark et al., 2001; Kostenis et al., 2005). A specific receptor for renin and its precursor prorenin (prorenin/renin receptor, PRR) may be of particular interest for the brain RAS (Nguyen et al., 2002). High levels of PRR were found in heart, brain, placenta and adipocytes, and lower level of expression in other tissues (Nguyen, 2011). PRR play a dual function (Nguyen and Contrepas, 2008; Shan et al., 2008): (i) Ang II-mediated effects; after binding to PRR, the catalytic activity of renin to hydrolyze angiotensinogen into angiotensin increases by about 4–5 times, and the precursor prorenin acquires catalytic properties similar to those of renin and (ii) Ang II-independent actions; PRR trigger their own signaling pathway that leads to pro-oxidative effects similar to those induced by activation of AT1 receptors.

## The Brain RAS

Initially, it was considered that the effects of RAS in the CNS were a consequence of the activity of the circulating RAS, acting through the circumventricular organs, on neurons regulating blood pressure and sodium and water homeostasis (Phillips and de Oliveira, 2008), because active components of the RAS, particularly Ang II, do not cross the blood-brain barrier (Harding et al., 1988). However, a local and independent RAS has now been identified in the brain. Astrocytes are the major source of brain angiotensinogen (Stornetta et al., 1988; Milsted et al., 1990), with only a small contribution from neurons (Kumar et al., 1988; Thomas et al., 1992).

Different RAS components were observed in several brain regions. In the basal ganglia, particularly in the nigrostriatal dopaminergic system, we have shown a local RAS using laser confocal microscopy and other methods such as *in situ* hybridization, laser microdissection and PCR or western blotting. In the substantia nigra, both AT1 and AT2 receptors were observed in dopaminergic neurons, astrocytes and microglia of rats (Rodriguez-Pallares et al., 2008), mice (Joglar et al., 2009), non-human primates (Valenzuela et al., 2010; Garrido-Gil et al., 2013b, 2017) and human brains (Garrido-Gil et al., 2013b). In addition, AT1 and AT2 receptors were observed in dopaminergic neurons and glial cells in primary cell cultures of the nigral region and several neuronal and glial cell lines (Rodriguez-Pallares et al., 2004, 2008; Joglar et al., 2009; Rodriguez-Perez et al., 2015a). In some studies, expression of AT1 receptors was not detected in microglial cells (Benicky et al., 2009). However, it is known that the level of microglial AT1 receptor expression is low in control (classically non-activated) microglia and is highly upregulated as part of the pro-inflammatory microglial response (Miyoshi et al., 2008; Rodriguez-Perez et al., 2015a; Dominguez-Meijide et al., 2017). Detection of AT1 expression may depend on the sensitivity threshold of the methodology used, and the level of pro-inflammatory activation of the microglial cells that are being analyzed. In addition, cytoplasmatic and membrane Nox subunits were located in dopaminergic neurons, astrocytes and microglia (Rodriguez-Pallares et al., 2007, 2008; Joglar et al., 2009).

## A Major Role for The NADPH-Oxidase Complex Activation in Polarization to Proinflammatory/M1 Phenotype

The complex NADPH-oxidase is a multi-component enzyme constituted by three cytosolic subunits (p40, p47 and p67) and at least two membrane subunits (gp91 and p22). The complex is inactive when the different subunits are spatially isolated. After stimulation, the complex is assembled and activated. In cells, mitochondria and the membrane NADPH-oxidase complex are the major sources of ROS (Babior, 2004). In addition, NADPH oxidase-derived ROS enhance production of ROS by mitochondria, intracellular iron uptake and other intracellular ROS sources (Cai, 2005). It is known that there is a ROS-mediated cross-talk signaling between the membrane Nox and mitochondria (Sheh et al., 2007; Alberici et al., 2009). This feed-forward mechanism enhances and sustains ROS production. Most cells appear to have Nox. However, Nox produces high levels of oxidants in phagocytes and low levels of ROS, particularly for signaling function, in other cell types (including neurons and glial cells). Initially, Nox-derived ROS may have been developed in cells as a signaling system, and then specialized as a defense system in macrophages (Babior, 2004). In phagocytes-neutrophiles and monocytes, Nox produces high levels of extracellular superoxide/ROS to eliminate invading microorganisms or unwanted cells (Babior, 2004; West et al., 2011). Superoxide induces tissue damage after being transformed into toxic species such as hydrogen peroxide and peroxynitrite, formed after reaction with NO. Furthermore, in the presence of Nox, iNOS oxidation of L-arginine (L-ARG) produces NO (MacMicking et al., 1997). ROS derived from Nox may also act indirectly by enhancing the production of proteases (Reeves et al., 2002). In macrophages and microglial cells, Nox-derived ROS also act on intracellular signaling pathways involved in microglial or macrophage activation and the release of proinflammatory signals (Qin et al., 2004).

## Ang II, Via AT1 Receptors, Is A Major Activator of The NADPH-Oxidase Complex. Role in Brain Diseases and Neurodegeneration

Abnormal upregulation of local Ang II induces OS and exacerbates inflammation. As indicated above, Ang II, via its AT1 receptor, is a major activator of the NADPH-oxidase complex (Zalba et al., 2001; Hoogwerf, 2010), and NADPH-dependent oxidases mediate several key aspects of OS and inflammatory processes that are involved in major degenerative diseases in peripheral tissues (Griendling et al., 2000; Münzel and Keaney, 2001). In the brain, the RAS has been involved in several disorders such as anxiety and stress (Peng et al., 2002), depressive illness (Saab et al., 2007), and alcohol intake (Maul et al., 2005). Inhibition of AT1 receptors has been reported to improve aging-related deficits in learning, spatial working memory and motor performance (Hellner et al., 2005; Kerr et al., 2005). AT1 receptor blockers and ACE inhibitors (ACEIs) have been shown to inhibit inflammation in the central nervous system (Platten et al., 2009; Stegbauer et al., 2009). Indeed, AT1 receptor antagonists exerted positive effects in several processes mediated by microglial activation and neuroinflammation, such as experimental models of multiple sclerosis (Platten et al., 2009; Stegbauer et al., 2009), Alzheimer’s disease (AD; Kehoe and Wilcock, 2007; Mogi and Horiuchi, 2009; Torika et al., 2016) and brain ischemia (Iwanami et al., 2010). Consistent with this, several clinical and genetic studies suggest a relationship between AD and RAS (reviewed in Savaskan, 2005; Kehoe and Wilcock, 2007; Mogi and Horiuchi, 2009). In a series of recent studies in animal models of PD, we have also observed that the brain RAS plays a major role in progression of dopaminergic degeneration.

**Figure 2:**
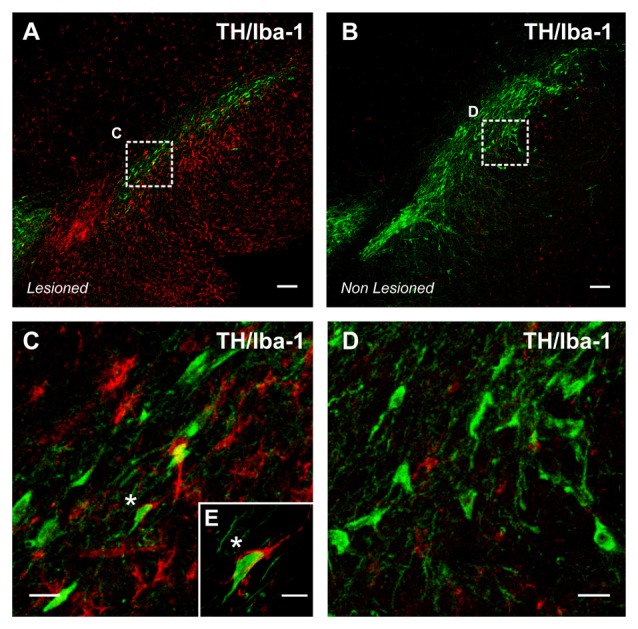
**Intense microglial activation in a degenerating rat substantia nigra** **(A)**, iba-1 positive red immunofluorescence. A high density of activated microglial cells is observed in the nigral region surrounding the dopaminergic cells. The degeneration of dopaminergic neurons (green; tyrosine hydroxylase, TH immunolabeled cells) was triggered by over expression of alpha-synuclein (not shown) after unilateral injection of adeno-associated viral vectors encoding alpha-synuclein. The contralateral non-lesioned substantia nigra of the same tissue section **(B)** shows scattered microglial cells in an apparent classical “non-activated” state. The areas squared in **(A,B)** are magnified in **(C–E)**, and a close interaction between a dopaminergic neuron and a microglial cell (asterisks) is shown in **(E)**. Scale bar: 100 μm **(A,B)**, 20 μm **(C,D)** and 10 μm **(E)**.

A number of experimental and clinical data suggest that neuroinflammation is a factor for the progression of dopaminergic neuron degeneration. Both PD patients (Ouchi et al., 2005; Ghadery et al., 2017) and PD animal models (Cicchetti et al., 2002; Rodriguez-Pallares et al., 2007) show enhanced microglial reaction in the substantia nigra and striatum (Figure 2). It was initially considered that the increase in the microglial response was a consequence of the neuronal death to remove dead cells and debris, although this inflammatory process may also lead to additional damage of the surrounding neurons and progression of neurodegeneration, as reported for several autoimmune diseases (Vowinckel et al., 1997). However, it has been shown that microglial response and Nox activation are major factors for triggering neuronal degeneration, acting synergistically with different pathogenic factors to induce neurodegeneration at early stages of the disease (Gao et al., 2003; Wu et al., 2003). In different PD models, we observed that Ang II increased the neurotoxic effect of low doses of dopaminergic neurotoxins, and that administration of ACE inhibitors (Lopez-Real et al., 2005; Muñoz et al., 2006) or AT1 receptor antagonists (Rey et al., 2007; Rodriguez-Pallares et al., 2008; Joglar et al., 2009) induced significant protection of dopaminergic neurons and reduction in neurotoxin-induced levels of protein oxidation and lipid peroxidation (Sánchez-Iglesias et al., 2007). Nox inhibitors also blocked the enhancing effect of Ang II on dopaminergic neurodegeneration, which confirmed that Nox activation and Nox-induced ROS play a major role in the Ang II-induced increase in neuronal loss (Rey et al., 2007; Rodriguez-Pallares et al., 2008; Joglar et al., 2009). In summary (Labandeira-Garcia et al., 2013; Labandeira-García et al., 2014), we suggest that Ang II acts by a double mechanism; in dopaminergic neurons (i.e., CNS resident cells), Ang II acts on AT1 receptors to produce low levels of intraneuronal ROS by activation of neuronal Nox. In addition, Ang II acts on microglia (i.e., inflammatory cells), and activation of the microglial AT1/ Nox axis induces the generation of high levels of superoxide and superoxide-derived ROS that, after being released to the extracellular medium, may lead to neuronal damage. In addition, a small amount of microglial ROS is used as a microglial second messenger in intracellular pathways that regulate the inflammatory response (Babior, 2004; Qin et al., 2004). Consistent with this, overactivation of the Ang II/AT1/Nox axis enhances vulnerability of dopaminergic neurons and synergistically contributes to initiation and progression of the disease. The role of brain RAS in dopaminergic neuron death and possibly in PD has also been shown by a number of studies from different research groups (Grammatopoulos et al., 2007; Zawada et al., 2011; Sonsalla et al., 2013).

## Overactivation of The Ang II/At1/NADPH-Oxidase Axis in Aging

Aging is the major risk factor for neurodegenerative diseases, and particularly for PD and AD (McCormack et al., 2004; Collier et al., 2007). Aged tissues, including brain tissue, are characterized by a proinflammatory, pro-oxidant state that leads to exacerbated responses to lesions and enhanced vulnerability to neurodegeneration (Csiszar et al., 2003; Choi et al., 2010). Consistent with this, age-related diseases such as hypertension, diabetes and atherosclerosis show increased NADPH-oxidase activity (Griendling et al., 2000; Mehta and Griendling, 2007). It is known that tissue Ang II, acting on AT1 receptors, plays a major role in inflammatory processes that induce age-related degenerative changes (Heymes et al., 1998; Basso et al., 2005). In normal non-pathological states, there is a tight regulation of Ang II-induced ROS (de Cavanagh et al., 2004; Garrido and Griendling, 2009). However, a marked hyperactivity of the Ang/AT1/Nox axis has been observed in aged tissues (Min et al., 2009; Cassis et al., 2010). Consistent with this, mice null for AT1 receptors showed longevity, which was related to a decrease in OS together with an increase in expression of the prosurvival gene sirtuin 3 and mitochondrial protection (Benigni et al., 2009; de Cavanagh et al., 2011; Valenzuela et al., 2016). Furthermore, AT1 receptor deletion decreased age-related progression of atherosclerosis (Umemoto, 2008).

**Figure 3:**
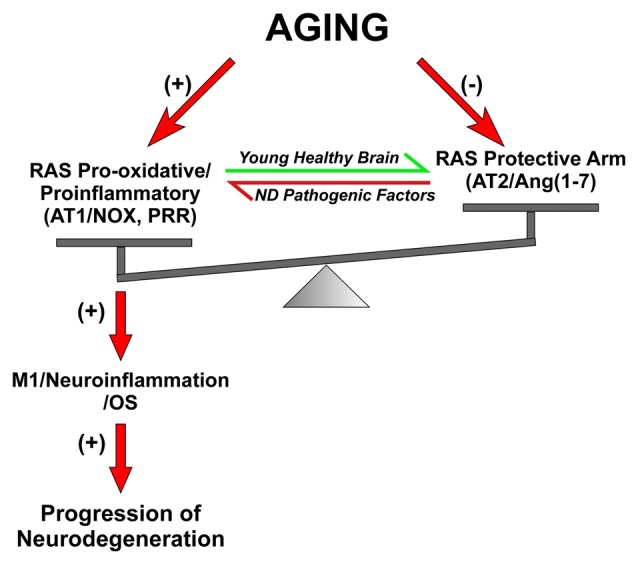
**Dysregulation of brain RAS with aging and other pathogenic factors.** In young and healthy brains the balance between the RAS pro-oxidative/proinflammatory and protective arms are tight regulated for physiological functions. Aging is associated with overactivation of the Ras pro-oxidative/proinflammatory arm and lack of compensatory upregulation of the protective arm, which leads to a pro-oxidative pro-inflammatory state and increased vulnerability to neurodegeneration. Other pathogenic factors involved in triggering neurodegeneration may also increase the activity of the RAS pro-inflammatory axis. Abbreviations: Ang, angiotensin; AT1, angiotensin type 1 receptor; AT2, angiotensin type 2 receptor; ND, neurodegenerative; Nox, NADPH-oxidase; OS, oxidative stress; PRR, prorenin/renin receptor.

We observed that the increase in AT1/Nox activity in the substantia nigra of aged rats plays a major role in the increase in vulnerability of dopaminergic neurons with aging (Figure 3). In aged animals, neurotoxins induced higher levels of dopaminergic neuron loss than in young animals (Sugama et al., 2003; McCormack et al., 2004), and nigral RAS was involved in this effect (Villar-Cheda et al., 2012b). A significant increase in Nox activity and levels of the pro-inflammatory cytokines IL-1β and TNF-α was observed in aged rats, which revealed a pro-oxidative and pro-inflammatory state in the aged substantia nigra. Aged rats also showed upregulation of AT1 receptor expression and down-regulation of AT2 receptor expression, which was inhibited by administration of the AT1 receptor blocker candesartan. The aging-related increase in AT1 receptors may lead to the increase in the Nox-derived OS and dopaminergic cell vulnerability to degeneration. In aged rats, Nox activation is further increased by the lack of the compensatory upregulation of AT2 receptors (see below), which was observed in young rats after upregulation of AT1 receptor signaling (Villar-Cheda et al., 2010). Aging-related loss of striatal D2 and D1 receptors (Wang et al., 1998; Ishibashi et al., 2009) and alteration of the dopaminergic system (Kubis et al., 2000; Collier et al., 2007; Cruz-Muros et al., 2009) have been observed in experimental models and human brains. It is also known that Ang II, via AT1 receptors, increases the release of dopamine (Mendelsohn et al., 1993; Brown et al., 1996; Dominguez-Meijide et al., 2014). Therefore, the upregulation of the Ang II/AT1/Nox axis that we observed in aged rats may be a compensatory change to counteract the decreased levels of dopamine or dopamine receptors (Villar-Cheda et al., 2012b, 2014). However, in addition to the dopaminergic down-regulation, other factors are probably involved in the aging-related upregulation of AT1/Nox activity, as aging has been shown to be associated with overactivation of the AngII/AT1/Nox axis in different tissues (Thompson et al., 2000; Min et al., 2009; Cassis et al., 2010).

## The Role of Other Angiotensin Receptors in Regulation of Microglial Polarization

**Figure 4:**
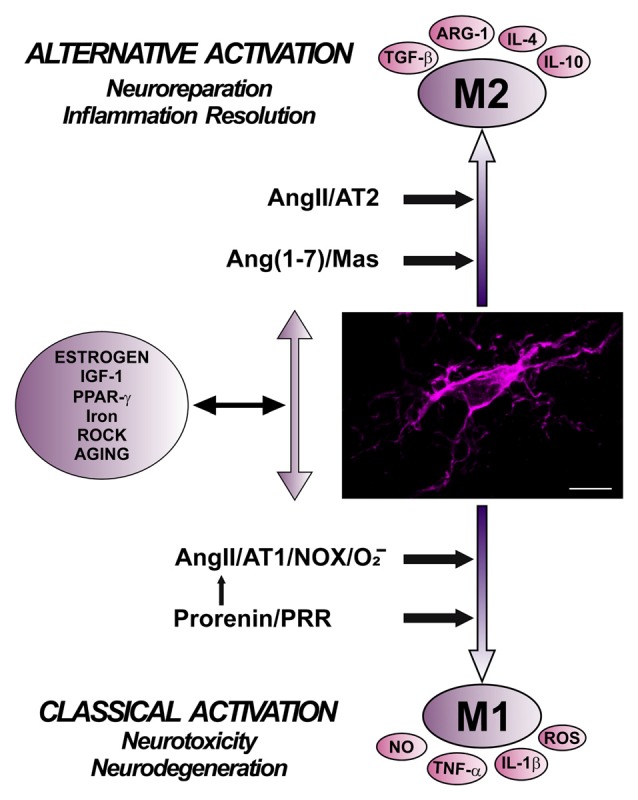
**Role of different angiotensin receptors in regulation of microglial polarization, and interactions with other microglial polarization regulators.** Angiotensin II, via AT1 receptor and Nox activation, and prorenin/renin, via PRR, induce pro-inflammatory effects and M1 phenotype, which is associated to increased levels of ROS, NO, TNF-α and IL-1β. These effects are counteracted by a RAS opposite arm constituted by angiotensin II/AT2 receptor signaling and Angiotensin 1–7/MasR signaling, which promote anti-inflammatory effects and M2 phenotype, which is also associated to TGF-β, IL-4, IL-10, and Arg-1 overexpression. In addition, there are interactions between RAS and different compounds involved in neuroinflammation and microglial polarization, such as estrogens, ROCK, IGF1, TNF-α, iron, PPARγ, and toll-like receptors. Abbreviations: Ang, angiotensin; ARG-1, arginase 1; AT1, angiotensin type 1 receptor; AT2, angiotensin type 2 receptor; IGF1, insulin-like growth factor-1; IL, interleukin; MasR, Mas receptor; NO, nitric oxide; Nox, NADPH-oxidase; O_2_^−^, superoxide; PPARγ, peroxisome proliferator-activated receptor gamma; PRR, prorenin/renin receptor; ROCK, Rho kinase; ROS, reactive oxygen species; TGF-β, transforming growth factor β; TNF-α tumor necrosis factor α. Scale bar: 7.5 μm.

Level of Nox activation has been suggested to be a major factor for regulation of the shift between proinflammatory/M1 and M2/immunoregulatory microglial phenotypes, so that Nox activation promotes proinflammatory and suppress the immunoregulatory phenotype (Choi et al., 2012). Consistent with this, AT1 receptor antagonists reduce proinflammatory microglia activation and promote immunoregulatory microglia polarization (Saavedra, 2012; Rodriguez-Perez et al., 2016; Torika et al., 2016). The classical Ang II/AT1/Nox pro-oxidative and pro-inflammatory axis plays a major role in the RAS effects both in the brain and peripheral tissues. However, it is now known that these effects are regulated by a RAS opposite arm constituted by Ang II/AT2 receptor signaling and Ang1–7/MasR signaling. A number of recent data suggest that activation of AT2 and/or MasRs plays an anti-oxidative/anti-inflammatory role. In addition, activation of PRR appears to contribute to activation of proinflammatory phenotype. However, the mechanisms involved in the interaction between different RAS receptors signaling to regulate microglial polarization and neuroinflammation are still unclear (Figure 4).

AT2 receptors counteract the effects of AT1 receptor activation and promote the M2/immunoregulatory phenotype. Reciprocal interaction between AT1 and AT2 receptors may regulate the effect of Ang II on cells (Sohn et al., 2000). Several studies in different types of cells have shown that AT2 activation decreases AT1 expression and NADPH-oxidase activation, leading to decrease in inflammatory responses (Jin et al., 2012; Yang et al., 2012; Lu et al., 2015). However, the intracellular pathways responsible for the interactions have not been clarified, and several mechanisms may be involved (Wu et al., 2004; Rompe et al., 2010; Yang et al., 2012; Dhande et al., 2013). Consistent with this, treatment with AT2 agonists led to reduction in the inflammatory responses, which were increased in AT2 receptor KO mice (Dhande et al., 2013; Iwanami et al., 2015). In the nigrostriatal system of young animals, we observed that upregulation of AT1 receptors was accompanied by a simultaneous and apparently compensatory upregulation of AT2 receptors (Villar-Cheda et al., 2010). In the nigra and striatum of aged rats, there was an increase in the expression of AT1 receptors and Nox activation; however, there was a lack of compensatory increase in AT2 expression (Villar-Cheda et al., 2010, 2012b). In aged animals, a decrease in AT2 receptor expression may lead to further increase in the pro-oxidative, pro-inflammatory effects and neuron vulnerability induced by activation of upregulated AT1 receptors.

MasRs, together with AT2 receptors, are considered major components of the RAS protective arm, which mediates actions opposing to AT1 (Bader, 2013; Villela et al., 2015). The ACE2 transforms Ang II into the heptapeptide Ang (1–7), which activates MasRs. It was observed that Mas-protooncogene is primarily expressed in the brain (Bunnemann et al., 1990; Von Bohlen und Halbach et al., 2000), and MasRs were identified in neurons, astrocytes and glial cells (Gallagher et al., 2006; Liu et al., 2016). Several recent studies suggest that activation of the Ang (1–7)/Mas axis inhibits the M1 inflammatory response in microglial cells and peripheral macrophages (Hammer et al., 2016; Liu et al., 2016; Tao et al., 2016). However, the cellular mechanisms involved in these effects remain to be clarified.

PRRs have been identified in neurons, astrocytes and microglial cells in rodents and primates, including humans (Valenzuela et al., 2010; Garrido-Gil et al., 2013b). PRR, together with AT1 receptors, promote the inflammatory response and the M1/proinflammatory phenotype (Shi et al., 2014; Zhu et al., 2015). The mechanisms involved in interactions of PRR and other RAS receptors to regulate microglial polarization remain to be investigated. The peptide Ang IV and its receptor have been involved in a number of functions in the CNS (Wright et al., 2015). There are a few data on possible involvement of Ang IV in inflammatory responses in peripheral tissues (Esteban et al., 2005; Kong et al., 2015). However, there are no data on the involvement of Ang IV in the neuroinflammatory response.

## Interactions Between RAS and Other Microglial Polarization Regulators

A number of studies have shown interactions or mutual regulation between RAS and different compounds involved in neuroinflammation and microglial polarization, such as estrogens, Rho kinase (ROCK), insulin-like growth factor-1 (IGF-1), TNF-α, iron, peroxisome proliferator-activated receptor gamma (PPARγ), and TLRs.

Estrogens protect neurons by mechanisms that are still unclear. However, modulation of the glial neuroinflammatory response plays a major role in the estrogen-induced neuroprotection (Morale et al., 2006; Suzuki et al., 2007; Vegeto et al., 2008). In several recent studies using PD models, we observed that estrogens modulate brain RAS and neuroinflammation acting both on astrocytes and microglia (Rodriguez-Perez et al., 2010, 2012, 2015b; Labandeira-Garcia et al., 2016). We observed that activation of microglial estrogen receptor (ER) β with the agonist 2,3-Bis-4-hydroxyphenyl-propionitrile (DPN)) inhibited the Ang II-induced increase in levels of several major mediators of the microglial inflammatory response such as IL-1β and ROCK (Villar-Cheda et al., 2012a; Rodriguez-Perez et al., 2013, 2015a).

RhoA/ROCK is a major modulator of the actin cytoskeleton, which regulates migration of microglia and other inflammatory cells (Yan et al., 2012; Labandeira-Garcia et al., 2015) into lesioned areas (Greenwood et al., 2003; Honing et al., 2004). RhoA/ROCK induces changes in the actin cytoskeleton necessary for cell motility, process retraction and cell spreading, which characterize activation of inflammatory cells, including microglia (Bernhart et al., 2010). Activation of the microglial RhoA/ROCK pathway plays a major role in the effect of Ang II/AT1/Nox axis on microglial polarization and neurodegeneration. This was shown in rodents (Barcia et al., 2012; Villar-Cheda et al., 2012a), and confirmed using mesencephalic cultures lacking microglial cells (Villar-Cheda et al., 2012a; Borrajo et al., 2014b). During Ang II-induced microglial activation, a crosstalk signaling between Nox and ROCK has been observed: Ang II-induced Nox activation led to superoxide production, NF-κB translocation and Rho-kinase activation. In addition, Rho-kinase activation was involved in regulation of NADPH-oxidase activation via p38 mitogen-activated protein kinase. Moreover, Rho-kinase activation, via NF-κB, upregulated angiotensin type-1 receptor expression in microglial cells through a feed-forward mechanism (Rodriguez-Perez et al., 2015a).

For many years, IGF-1 was considered a cytoprotective factor. However, it has also been suggested that IGF-1 may be detrimental to health, and that reduced IGF-1 levels lead to prolonged life (Tao et al., 2007; Suh et al., 2008). Local brain IGF-1 is produced by neurons and glial cells (Quesada et al., 2007; Suh et al., 2013), but the effects of IGF-1 in the brain and particularly in the aged brain are unclear (Bartke et al., 2003; Brown-Borg, 2007). Inhibitory effects on the neuroinflammatory response (Nadjar et al., 2009), improvement in mitochondrial function (Puche et al., 2008), inhibition of OS and Sirtuin-1 activation (Vinciguerra et al., 2009; Tran et al., 2014) have been suggested as possible mechanisms. Interestingly, the brain RAS has been shown to be involved in neuroinflammation (Borrajo et al., 2014a; Rodriguez-Perez et al., 2015a), mitochondrial function (Zawada et al., 2011; Rodriguez-Pallares et al., 2012) and Sirtuin-1 activity (Diaz-Ruiz et al., 2015). Recently, we observed a reciprocal regulation between IGF-1 and RAS (Rodriguez-Perez et al., 2016): IGF-1 inhibited AT1/Nox activity in neurons and glial cells (i.e., it decreased AT1, increased AT2 and decreased angiotensinogen expression); conversely, Ang II—via AT1 receptors—increased the levels of IGF-1 in microglial cells, while activation of AT2 receptors decreased IGF-1 levels. We observed that Ang II, via AT1 receptors, induced an increase in markers of the proinflammatory phenotype, which was blocked by treatment with IGF-1, suggesting that induction of microglial IGF-1 by Ang II and other OS and pro-inflammatory inducers may play a major role in repressing the M1-neurotoxic phenotype and transition to an M2-repair/regenerative phenotype. Our study showed that IGF-1 and the local RAS interact to inhibit or activate neuroinflammation (i.e., transition from the M1/proinflammatory to the M2/immunoregulatory phenotype). However, in brains from aged rats, particularly in the substantia nigra, we observed increased Ang II/AT1/Nox activity, while IGF-I levels remained decreased. The loss of the mechanism that upregulates IGF-1 to inhibit the AT1/Nox axis (Rodriguez-Perez et al., 2016) may lead to the loss of capacity of microglia to undergo M2/immunoregulatory activation and the pro-oxidative and pro-inflammatory state that characterizes the aged brain, and particularly the aged substantia nigra (Rey et al., 2007; Villar-Cheda et al., 2012b). IGF-1 and the local RAS may interact to inhibit or activate neuroinflammation and this important mechanism may be impaired in aged animals.

TNF-α is a pro-inflammatory cytokine that is involved in the pathogenesis of several neurodegenerative diseases (Hofman et al., 1989; Fillit et al., 1991; Mogi et al., 1994). In particular, dopaminergic neurons are highly vulnerable to TNF-α (McGuire et al., 2001; Qin et al., 2007; Harms et al., 2012). In primary mesencephalic cultures, we have recently observed that administration of low doses of the neurotoxin MPP^+^ led to significant dopaminergic cell death, which was increased by co-treatment with Ang II and decreased by administration of TNF-α blockers (Borrajo et al., 2014a). Furthermore, treatment with Ang II induced a marked increase in levels of TNF-α in primary mesencephalic cultures, which was blocked by administration of AT1 receptor antagonists, NADPH-oxidase inhibitors and NFK-β blockers. In cultures lacking microglial cells, however, levels of TNF-α were not significantly affected by administration of Ang II. We also observed that AT1 receptors, NADPH-oxidase, Rho-kinase and NFK-β were involved in the release of TNF-α by microglia (Borrajo et al., 2014a). These results revealed an important functional interaction between Ang II and TNF-α, and also that microglial TNF-α is a major mediator of Ang II-induced neuroinflammation and dopaminergic neurodegeneration.

Microglia play a major role in iron storage and homeostasis, and Ang II modulates microglial ferritin/iron storage levels and the neuroinflammatory response (Garrido-Gil et al., 2013a). Microglial ferritin levels (i.e., iron storage by ferritin) change in response to OS and microglial activation, and the release of iron from ferritin in activated microglia may enhance the oxidative damage derived from the proinflammatory microglial response (Mehlhase et al., 2006). Consistent with this, we observed in PD models that intense RAS overactivity and OS induced an increase in microglial labile iron and a decrease in ferritin levels. Although some data are controversial (Zecca et al., 2001), it is usually accepted that iron and ferritin increase in the substantia nigra with aging (Dusek et al., 2012). Aging-related increase in microglial ferritin has been associated to a less efficient control of iron homeostasis by senescent microglia (Lopes et al., 2008). In several studies in aged rats, we observed hyperactivity of the AT1/Nox axis in the substantia nigra, together with an increase in the levels of iron and ferritin, which were inhibited by AT1 receptor blockers such as candesartan. This suggests that overactivity of the Ang II/AT1/Nox axis plays a key role in aging-related iron and ferritin increase in the substantia nigra (Garrido-Gil et al., 2013a). The aging-associated upregulation of Ang II/AT1/Nox activity may lead to upregulation of iron storage by microglia and induce a pro-oxidative pro-inflammatory state and higher vulnerability of dopaminergic cells to degeneration with aging (Collier et al., 2007; Villar-Cheda et al., 2009, 2012b).

PPAR-γ is a member of a group of nuclear receptors (PPARs), which have been related to regulation of macrophage and adipocyte differentiation, as well as glucose and lipid metabolism and energy homeostasis. PPAR-γ receptors are also involved in inhibition of expression of different inflammatory cytokines and downregulation of the inflammatory process (Jiang et al., 1998; Ricote et al., 1998). PPAR-γ receptors are also involved in regulation of microglial activation and suppression of the proinflammatory phenotype (Bernardo et al., 2000; Mrak and Landreth, 2004). It was also shown that activation of the PPAR-γ induces the microglial immunoregulatory/M2 phenotype (Benoit et al., 2008; Rajaram et al., 2010). PPARγ agonists have been suggested as promising therapy for neurodegenerative diseases enhanced by neuroinflammation such as AD and PD (Schintu et al., 2009; Mandrekar-Colucci et al., 2012). Several studies have shown that AT1 receptor antagonists activate PPARγ. This has been related to pharmacological properties of some AT1 blockers such as telmisartan (Xu et al., 2015). However, studies using AT1 null mice revealed that blockage of AT1 receptors, independently of the pharmacological properties of the antagonists, also inhibits neuroinflammation and neurodegeneration (Garrido-Gil et al., 2012). Inhibition of AT1 with antagonists (ARBs), in addition to inhibition of proinflammatory polarization (see above), leads to activation of PPAR-γ and promotes the immunoregulatory phenotype by a double mechanism: by a pharmacological AT1-independent PPARγ agonistic effect (with more or less activation potency depending on the type of ARB), and by a direct effect of the blockage of the AT1 itself, which also induces PPARγ activation (Garrido-Gil et al., 2012). Among ARBS, the role of PPARγ activation in anti-inflammatory effects is particularly important for telmisartan, both at peripheral and CNS levels (Garrido-Gil et al., 2012; Pang et al., 2012; Wang et al., 2014; Villapol and Saavedra, 2015).

TLRs such as TLR2 and TLR4 are known to mediate classical microglial activation and neuroinflammation (Rietdijk et al., 2016). An increasing number of studies suggest a functional interaction or crosstalk between AT1 receptors and TLR4 and/or TLR2 (Biancardi et al., 2016). AT1 receptor antagonists inhibit TLRs in different immune effector cells both in peripheral tissues (Dasu et al., 2009; Cheng et al., 2011) and in brain microglia (Daniele et al., 2015; Biancardi et al., 2016). Ang II, via AT1, has been shown to increase TLR4 expression and Ang II-mediated ROS production and Ang II-induced microglial activation was blunted in TLR4 deficient mice (Rietdijk et al., 2016). The mechanisms involved in Ang II/AT1/TLRs interactions remain unknown.

Several recent studies suggest a pivotal role of metabolic reprogramming in the regulation of the innate inflammatory response (Galván-Peña and O’Neill, 2014; Tannahill et al., 2015). In the context of the peripheral immune cells, a shift in the cellular metabolism from oxidative phosphorylation to a erobic glycolysis for energy production favors the polarization toward a proinflammatory phenotype. More recently, the link between polarization and mitochondrial energy metabolism has been considered in microglia (see for review Orihuela et al., 2016). Interestingly, we have recently shown that an intracellular RAS, including AT1 and AT2 receptors, exists in brain mitochondria (Valenzuela et al., 2016). Activation of mitochondrial AT1 receptors regulates (by activation of mitochondrial Nox4) levels of superoxide and increases mitochondrial respiration. Mitochondrial AT2 receptors are much more abundant and downregulate mitochondrial respiration via NO production. Interestingly, altered expression of mitochondrial angiotensin receptors was observed in aged rats. Altered expression of AT1 and AT2 receptors with aging may contribute to mitochondrial dysfunction, neuroinflammation and neurodegeneration.

## Conclusion

Dysregulation of brain RAS plays a major role in aging-related changes and neurodegeneration by exacerbation of OS and neuroinflammation, which may be attenuated by pharmacological manipulation of RAS components. Angiotensin II (via AT1 receptor and Nox activation) and prorenin/renin (via PRR) induce pro-inflammatory effects and M1 microglial phenotype. These effects are counteracted by a RAS opposite arm constituted by angiotensin II/AT2 receptor signaling and Angiotensin 1–7/MasR signaling, which promote anti-inflammatory effects and M2 microglial phenotype. In addition, there are interactions between RAS and different compounds involved in neuroinflammation and microglial polarization, such as estrogens, ROCK, IGF1, TNF-α, iron, PPARγ and TLRs. Pharmacological manipulation of brain RAS components may be an interesting therapeutical approach for neurodegenerative diseases and aging-related processes in which OS and neuroinflammation play a major role.

## Author Contributions

All authors have contributed to this work and approved its final version for submission. JLL-G developed the idea for this review article and wrote the manuscript. AIR-P prepared the figures and was involved in literature review, and revision of the manuscript. JLL prepared the viral vectors used for activation of microglia in Figure 2, and revised the manuscript. PG-G, JR-P and MJG were involved in literature review, and preparation of the manuscript.

## Conflict of Interest Statement

The authors declare that the research was conducted in the absence of any commercial or financial relationships that could be construed as a potential conflict of interest.

## References

[B1] Alberici L. C. Oliveira H. C. Paim B. A. Mantello C. C. Augusto A. C. Zecchin K. G.. (2009). Mitochondrial ATP-sensitive K^+^ channels as redox signals to liver mitochondria in response to hypertriglyceridemia. Free Radic. Biol. Med. 47, 1432–1439. 10.1016/j.freeradbiomed.2009.08.013 19703550

[B2] Albiston A. L. McDowall S. G. Matsacos D. Sim P. Clune E. Mustafa T.. (2001). Evidence that the angiotensin IV (AT_4_) receptor is the enzyme insulin-regulated aminopeptidase. J. Biol. Chem. 276, 48623–48626. 10.1074/jbc.c100512200 11707427

[B3] Alliot F. Godin I. Pessac B. (1999). Microglia derive from progenitors, originating from the yolk sac, and which proliferate in the brain. Dev. Brain Res. 117, 145–152. 10.1016/s0165-3806(99)00113-3 10567732

[B4] Babior B. M. (2004). NADPH oxidase. Curr. Opin. Immunol. 16, 42–47. 10.1016/j.coi.2003.12.001 14734109

[B5] Bacani C. Frishman W. H. (2006). Chymase: a new pharmacologic target in cardiovascular disease. Cardiol. Rev. 14, 187–193. 10.1097/01.crd.0000195220.62533.c5 16788331

[B6] Bader M. (2013). ACE2, angiotensin-(1–7), and Mas: the other side of the coin. Pflugers Arch. 465, 79–85. 10.1007/s00424-012-1120-0 23463883

[B7] Balakumar P. Jagadeesh G. (2014). A century old renin-angiotensin system still grows with endless possibilities: AT1 receptor signaling cascades in cardiovascular physiopathology. Cell. Signal. 26, 2147–2160. 10.1016/j.cellsig.2014.06.011 25007996

[B8] Barcia C. Ros C. M. Annese V. Carrillo-de Sauvage M. A. Ros-Bernal F. Gómez A.. (2012). ROCK/Cdc42-mediated microglial motility and gliapse formation lead to phagocytosis of degenerating dopaminergic neurons *in vivo*. Sci. Rep. 2:809. 10.1038/srep00809 23139861 PMC3492875

[B9] Bartke A. Chandrashekar V. Dominici F. Turyn D. Kinney B. Steger R.. (2003). Insulin-like growth factor 1 (IGF-1) and aging: controversies and new insights. Biogerontology 4, 1–8. 10.1023/A:1022448532248 12652183

[B10] Basso N. Paglia N. Stella I. de Cavanagh E. M. Ferder L. del Rosario Lores Arnaiz M.. (2005). Protective effect of the inhibition of the renin-angiotensin system on aging. Regul. Pept. 128, 247–252. 10.1016/j.regpep.2004.12.027 15837534

[B11] Benicky J. Sánchez-Lemus E. Pavel J. Saavedra J. M. (2009). Anti-inflammatory effects of angiotensin receptor blockers in the brain and the periphery. Cell. Mol. Neurobiol. 29, 781–792. 10.1007/s10571-009-9368-4 19259805 PMC2718067

[B12] Benigni A. Corna D. Zoja C. Sonzogni A. Latini R. Salio M.. (2009). Disruption of the Ang II type 1 receptor promotes longevity in mice. J. Clin. Invest. 119, 524–530. 10.1172/JCI36703 19197138 PMC2648681

[B13] Benoit M. Desnues B. Mege J. L. (2008). Macrophage polarization in bacterial infections. J. Immunol. 181, 3733–3739. 10.4049/jimmunol.181.6.3733 18768823

[B14] Bernardo A. Levi G. Minghetti L. (2000). Role of the peroxisome proliferator-activated receptor-γ (PPAR-γ) and its natural ligand 15-deoxy-∆^12, 14^-prostaglandin J_2_ in the regulation of microglial functions. Eur. J. Neurosci. 12, 2215–2223. 10.1046/j.1460-9568.2000.00110.x 10947800

[B15] Bernhart E. Kollroser M. Rechberger G. Reicher H. Heinemann A. Schratl P.. (2010). Lysophosphatidic acid receptor activation affects the C13NJ microglia cell line proteome leading to alterations in glycolysis, motility, and cytoskeletal architecture. Proteomics 10, 141–158. 10.1002/pmic.200900195 19899077 PMC4060044

[B17] Biancardi V. C. Stranahan A. M. Krause E. G. de Kloet A. D. Stern J. E. (2016). Cross talk between AT1 receptors and Toll-like receptor 4 in microglia contributes to angiotensin II-derived ROS production in the hypothalamic paraventricular nucleus. Am. J. Physiol. Heart Circ. Physiol. 310, H404–H415. 10.1152/ajpheart.00247.2015 26637556 PMC4796625

[B18] Borrajo A. Rodriguez-Perez A. I. Diaz-Ruiz C. Guerra M. J. Labandeira-Garcia J. L. (2014a). Microglial TNF-α mediates enhancement of dopaminergic degeneration by brain angiotensin. Glia 62, 145–157. 10.1002/glia.22595 24272709

[B19] Borrajo A. Rodriguez-Perez A. I. Villar-Cheda B. Guerra M. J. Labandeira-Garcia J. L. (2014b). Inhibition of the microglial response is essential for the neuroprotective effects of Rho-kinase inhibitors on MPTP-induced dopaminergic cell death. Neuropharmacology 85, 1–8. 10.1016/j.neuropharm.2014.05.021 24878243

[B20] Brown D. C. Steward L. J. Ge J. Barnes N. M. (1996). Ability of angiotensin II to modulate striatal dopamine release via the AT1 receptor *in vitro* and *in vivo*. Br. J. Pharmacol. 118, 414–420. 10.1111/j.1476-5381.1996.tb15418.x 8735646 PMC1909619

[B21] Brown-Borg H. M. (2007). Hormonal regulation of longevity in mammals. Ageing Res. Rev. 6, 28–45. 10.1016/j.arr.2007.02.005 17360245 PMC1978093

[B22] Bunnemann B. Fuxe K. Metzger R. Mullins J. Jackson T. R. Hanley M. R.. (1990). Autoradiographic localization of mas proto-oncogene mRNA in adult rat brain using *in situ* hybridization. Neurosci. Lett. 114, 147–153. 10.1016/0304-3940(90)90063-f 2203997

[B23] Butovsky O. Jedrychowski M. P. Moore C. S. Cialic R. Lanser A. J. Gabriely G.. (2014). Identification of a unique TGF-β-dependent molecular and functional signature in microglia. Nat. Neurosci. 17, 131–143. 10.1038/nn.3599 24316888 PMC4066672

[B24] Cai H. (2005). NAD(P)H oxidase–dependent self-propagation of hydrogen peroxide and vascular disease. Circ. Res. 96, 818–822. 10.1161/01.res.0000163631.07205.fb 15860762

[B25] Carson M. J. Doose J. M. Melchior B. Schmid C. D. Ploix C. C. (2006). CNS immune privilege: hiding in plain sight. Immunol. Rev. 213, 48–65. 10.1111/j.1600-065x.2006.00441.x 16972896 PMC2633103

[B26] Cassis P. Conti S. Remuzzi G. Benigni A. (2010). Angiotensin receptors as determinants of life span. Pflugers Arch. 459, 325–332. 10.1007/s00424-009-0725-4 19763608

[B27] Chabrashvili T. Kitiyakara C. Blau J. Karber A. Aslam S. Welch W. J.. (2003). Effects of ANG II type 1 and 2 receptors on oxidative stress, renal NADPH oxidase and SOD expression. Am. J. Physiol. Regul. Integr. Comp. Physiol. 285, R117–R124. 10.1152/ajpregu.00476.2002 12609817

[B28] Cheng X. W. Song H. Sasaki T. Hu L. Inoue A. Bando Y. K.. (2011). Angiotensin type 1 receptor blocker reduces intimal neovascularization and plaque growth in apolipoprotein E-deficient mice. Hypertension 57, 981–989. 10.1161/HYPERTENSIONAHA.110.168385 21464389 PMC3319395

[B29] Chhor V. Le Charpentier T. Lebon S. Oré M. V. Celador I. L. Josserand J.. (2013). Characterization of phenotype markers and neuronotoxic potential of polarised primary microglia *in vitro*. Brain Behav. Immun. 32, 70–85. 10.1016/j.bbi.2013.02.005 23454862 PMC3694309

[B31] Choi S.-H. Aid S. Kim H.-W. Jackson S. H. Bosetti F. (2012). Inhibition of NADPH oxidase promotes alternative and anti-inflammatory microglial activation during neuroinflammation. J. Neurochem. 120, 292–301. 10.1111/j.1471-4159.2011.07572.x 22050439 PMC3386526

[B30] Choi D.-Y. Zhang J. Bing G. (2010). Aging enhances the neuroinflammatory response and α-synuclein nitration in rats. Neurobiol. Aging 31, 1649–1653. 10.1016/j.neurobiolaging.2008.09.010 18986737

[B32] Cicchetti F. Brownell A. L. Williams K. Chen Y. I. Livni E. Isacson O. (2002). Neuroinflammation of the nigrostriatal pathway during progressive 6-OHDA dopamine degeneration in rats monitored by immunohistochemistry and PET imaging. Eur. J. Neurosci. 15, 991–998. 10.1046/j.1460-9568.2002.01938.x 11918659

[B33] Clark M. A. Diz D. I. Tallant E. A. (2001). Angiotensin-(1–7) downregulates the angiotensin II type 1 receptor in vascular smooth muscle cells. Hypertension 37, 1141–1146. 10.1161/01.hyp.37.4.1141 11304516

[B34] Collier T. J. Lipton J. Daley B. F. Palfi S. Chu Y. Sortwell C.. (2007). Aging-related changes in the nigrostriatal dopamine system and the response to MPTP in nonhuman primates: diminished compensatory mechanisms as a prelude to parkinsonism. Neurobiol. Dis. 26, 56–65. 10.1016/j.nbd.2006.11.013 17254792 PMC1899875

[B35] Colton C. A. (2009). Heterogeneity of microglial activation in the innate immune response in the brain. J. Neuroimmune Pharmacol. 4, 399–418. 10.1007/s11481-009-9164-4 19655259 PMC2773116

[B36] Cruz-Muros I. Afonso-Oramas D. Abreu P. Pérez-Delgado M. M. Rodriguez M. González-Hernández T. (2009). Aging effects on the dopamine transporter expression and compensatory mechanisms. Neurobiol. Aging 30, 973–986. 10.1016/j.neurobiolaging.2007.09.009 17976862

[B37] Csiszar A. Ungvari Z. Koller A. Edwards J. G. Kaley G. (2003). Aging-induced proinflammatory shift in cytokine expression profile in coronary arteries. FASEB J. 17, 1183–1185. 10.1096/fj.02-1049fje 12709402

[B38] Daniele S. G. Béraud D. Davenport C. Cheng K. Yin H. Maguire-Zeiss K. A. (2015). Activation of MyD88-dependent TLR1/2 signaling by misfolded α-synuclein, a protein linked to neurodegenerative disorders. Sci. Signal. 8:ra45. 10.1126/scisignal.2005965 25969543 PMC4601639

[B39] Dasu M. R. Riosvelasco A. C. Jialal I. (2009). Candesartan inhibits Toll-like receptor expression and activity both *in vitro* and *in vivo*. Atherosclerosis 202, 76–83. 10.1016/j.atherosclerosis.2008.04.010 18495130 PMC2676176

[B40] Davalos D. Grutzendler J. Yang G. Kim J. V. Zuo Y. Jung S.. (2005). ATP mediates rapid microglial response to local brain injury *in vivo*. Nat. Neurosci. 8, 752–758. 10.1038/nn1472 15895084

[B41] de Cavanagh E. M. Inserra F. Ferder L. (2011). Angiotensin II blockade: a strategy to slow ageing by protecting mitochondria? Cardiovasc. Res. 89, 31–40. 10.1093/cvr/cvq285 20819950

[B42] de Cavanagh E. M. Piotrkowski B. Fraga C. G. (2004). Concerted action of the renin-angiotensin system, mitochondria, and antioxidant defenses in aging. Mol. Aspects Med. 25, 27–36. 10.1016/j.mam.2004.02.006 15051314

[B43] Dhande I. Ali Q. Hussain T. (2013). Proximal tubule angiotensin AT2 receptors mediate an anti-inflammatory response via interleukin-10: role in renoprotection in obese rats. Hypertension 61, 1218–1226. 10.1161/HYPERTENSIONAHA.111.00422 23547236 PMC3709839

[B44] Diaz-Ruiz C. Rodriguez-Perez A. I. Beiroa D. Rodriguez-Pallares J. Labandeira-Garcia J. L. (2015). Reciprocal regulation between sirtuin-1 and angiotensin-II in the substantia nigra: implications for aging and neurodegeneration. Oncotarget 6, 26675–26689. 10.18632/oncotarget.5596 26384348 PMC4694944

[B45] Dominguez-Meijide A. Rodriguez-Perez A. I. Diaz-Ruiz C. Guerra M. J. Labandeira-Garcia J. L. (2017). Dopamine modulates astroglial and microglial activity via glial renin-angiotensin system in cultures. Brain Behav. Immun. 62, 277–290. 10.1016/j.bbi.2017.02.013 28232171

[B46] Dominguez-Meijide A. Villar-Cheda B. Garrido-Gil P. Sierrra-Paredes G. Guerra M. J. Labandeira-Garcia J. L. (2014). Effect of chronic treatment with angiotensin type 1 receptor antagonists on striatal dopamine levels in normal rats and in a rat model of Parkinson’s disease treated with L-DOPA. Neuropharmacology 76, 156–168. 10.1016/j.neuropharm.2013.07.016 23973568

[B47] Dusek P. Jankovic J. Le W. (2012). Iron dysregulation in movement disorders. Neurobiol. Dis. 46, 1–18. 10.1016/j.nbd.2011.12.054 22266337

[B48] Edwards J. P. Zhang X. Frauwirth K. A. Mosser D. M. (2006). Biochemical and functional characterization of three activated macrophage populations. J. Leukoc. Biol. 80, 1298–1307. 10.1189/jlb.0406249 16905575 PMC2642590

[B49] Esteban V. Ruperez M. Sánchez-López E. Rodriguez-Vita J. Lorenzo O. Demaegdt H.. (2005). Angiotensin IV activates the nuclear transcription factor-κB and related proinflammatory genes in vascular smooth muscle cells. Circ. Res. 96, 965–973. 10.1161/01.res.0000166326.91395.74 15831814

[B50] Fillit H. Ding W. H. Buee L. Kalman J. Altstiel L. Lawlor B.. (1991). Elevated circulating tumor necrosis factor levels in Alzheimer’s disease. Neurosci. Lett. 129, 318–320. 10.1016/0304-3940(91)90490-k 1745413

[B51] Franco R. Fernández-Suárez D. (2015). Alternatively activated microglia and macrophages in the central nervous system. Prog. Neurobiol. 131, 65–86. 10.1016/j.pneurobio.2015.05.003 26067058

[B52] Frank-Cannon T. C. Alto L. T. McAlpine F. E. Tansey M. G. (2009). Does neuroinflammation fan the flame in neurodegenerative diseases? Mol. Neurodegener. 4:47. 10.1186/1750-1326-4-47 19917131 PMC2784760

[B53] Fujisaka S. Usui I. Kanatani Y. Ikutani M. Takasaki I. Tsuneyama K.. (2011). Telmisartan improves insulin resistance and modulates adipose tissue macrophage polarization in high-fat-fed mice. Endocrinology 152, 1789–1799. 10.1210/en.2010-1312 21427223

[B54] Gallagher P. E. Chappell M. C. Ferrario C. M. Tallant E. A. (2006). Distinct roles for ANG II and ANG-(1–7) in the regulation of angiotensin-converting enzyme 2 in rat astrocytes. Am. J. Physiol. Cell Physiol. 290, C420–C426. 10.1152/ajpcell.00409.2004 16176966

[B55] Galván-Peña S. A. O’Neill L. A. (2014). Metabolic reprograming in macrophage polarization. Front. Immunol. 5:420. 10.3389/fimmu.2014.00420 25228902 PMC4151090

[B56] Ganong W. F. (1994). Origin of the angiotensin II secreted by cells. Proc. Soc. Exp. Biol. Med. 205, 213–219. 10.3181/00379727-205-43699a 8171042

[B57] Gao H. M. Liu B. Zhang W. Hong J. S. (2003). Critical role of microglial NADPH oxidase-derived free radicals in the *in vitro* MPTP model of Parkinson’s disease. FASEB J. 17, 1954–1956. 10.1096/fj.03-0109fje 12897068

[B58] Garrido A. M. Griendling K. K. (2009). NADPH oxidases and angiotensin II receptor signaling. Mol. Cell. Endocrinol. 302, 148–158. 10.1016/j.mce.2008.11.003 19059306 PMC2835147

[B59] Garrido-Gil P. Joglar B. Rodriguez-Perez A. I. Guerra M. J. Labandeira-Garcia J. L. (2012). Involvement of PPAR-γ in the neuroprotective and anti-inflammatory effects of angiotensin type 1 receptor inhibition: effects of the receptor antagonist telmisartan and receptor deletion in a mouse MPTP model of Parkinson’s disease. J. Neuroinflammation 9:38. 10.1186/1742-2094-9-38 22356806 PMC3298706

[B60] Garrido-Gil P. Rodriguez-Pallares J. Dominguez-Meijide A. Guerra M. J. Labandeira-Garcia J. L. (2013a). Brain angiotensin regulates iron homeostasis in dopaminergic neurons and microglial cells. Exp. Neurol. 250, 384–396. 10.1016/j.expneurol.2013.10.013 24184051

[B62] Garrido-Gil P. Valenzuela R. Villar-Cheda B. Lanciego J. L. Labandeira-Garcia J. L. (2013b). Expression of angiotensinogen and receptors for angiotensin and prorenin in the monkey and human substantia nigra: an intracellular renin-angiotensin system in the nigra. Brain Struct. Funct. 218, 373–388. 10.1007/s00429-012-0402-9 22407459 PMC3580133

[B61] Garrido-Gil P. Rodriguez-Perez A. I. Fernandez-Rodriguez P. Lanciego J. L. Labandeira-Garcia J. L. (2017). Expression of angiotensinogen and receptors for angiotensin and prorenin in the rat and monkey striatal neurons and glial cells. Brain Struct. Funct. [Epub ahead of print]. 10.1007/s00429-016-1357-z 28161727

[B63] Gautier E. L. Shay T. Miller J. Greter M. Jakubzick C. Ivanov S.. (2012). Gene-expression profiles and transcriptional regulatory pathways that underlie the identity and diversity of mouse tissue macrophages. Nat. Immunol. 13, 1118–1128. 10.1038/ni.2419 23023392 PMC3558276

[B64] Ghadery C. Koshimori Y. Coakeley S. Harris M. Rusjan P. Kim J.. (2017). Microglial activation in Parkinson’s disease using [^18^F]-FEPPA. J. Neuroinflammation 14:8. 10.1186/s12974-016-0778-1 28086916 PMC5234135

[B65] Ginhoux F. Greter M. Leboeuf M. Nandi S. See P. Gokhan S.. (2010). Fate mapping analysis reveals that adult microglia derive from primitive macrophages. Science 330, 841–845. 10.1126/science.1194637 20966214 PMC3719181

[B66] Gordon S. (2003). Alternative activation of macrophages. Nat. Rev. Immunol. 3, 23–35. 10.1038/nri978 12511873

[B67] Grammatopoulos T. N. Jones S. M. Ahmadi F. A. Hoover B. R. Snell L. D. Skoch J.. (2007). Angiotensin type 1 receptor antagonist losartan, reduces MPTP-induced degeneration of dopaminergic neurons in substantia nigra. Mol. Neurodegener. 2:1. 10.1186/1750-1326-2-1 17224059 PMC1783655

[B68] Greenwood J. Walters C. E. Pryce G. Kanuga N. Beraud E. Baker D.. (2003). Lovastatin inhibits brain endothelial cell Rho-mediated lymphocyte migration and attenuates experimental autoimmune encephalomyelitis. FASEB J. 17, 905–907. 10.1096/fj.02-1014fje 12626426 PMC3831156

[B69] Griendling K. K. Sorescu D. Ushio-Fukai M. (2000). NAD(P)H oxidase: role in cardiovascular biology and disease. Circ. Res. 86, 494–501. 10.1161/01.res.86.5.494 10720409

[B70] Hammer A. Yang G. Friedrich J. Kovacs A. Lee D. H. Grave K.. (2016). Role of the receptor Mas in macrophage-mediated inflammation *in vivo*. Proc. Natl. Acad. Sci. U S A 113, 14109–14114. 10.1073/pnas.1612668113 27872279 PMC5150410

[B71] Hamming I. Cooper M. E. Haagmans B. L. Hooper N. M. Korstanje R. Osterhaus A. D.. (2007). The emerging role of ACE2 in physiology and disease. J. Pathol. 212, 1–11. 10.1002/path.2162 17464936 PMC7167724

[B72] Harding J. W. Sullivan M. J. Hanesworth J. M. Cushing L. L. Wright J. W. (1988). Inability of [125I]Sar1, Ile8-angiotensin II to move between the blood and cerebrospinal fluid compartments. J. Neurochem. 50, 554–557. 10.1111/j.1471-4159.1988.tb02946.x 3335860

[B73] Harms A. S. Lee J. K. Nguyen T. A. Chang J. Ruhn K. M. Treviño I.. (2012). Regulation of microglia effector functions by tumor necrosis factor signaling. Glia 60, 189–202. 10.1002/glia.21254 21989628 PMC3232308

[B74] Harrison J. K. Jiang Y. Chen S. Xia Y. Maciejewski D. McNamara R. K.. (1998). Role for neuronally derived fractalkine in mediating interactions between neurons and CX3CR1-expressing microglia. Proc. Natl. Acad. Sci. U S A 95, 10896–10901. 10.1073/pnas.95.18.10896 9724801 PMC27992

[B75] Harry G. J. Kraft A. D. (2012). Microglia in the developing brain: a potential target with lifetime effects. Neurotoxicology 33, 191–206. 10.1016/j.neuro.2012.01.012 22322212 PMC3299893

[B76] Hellner K. Walther T. Schubert M. Albrecht D. (2005). Angiotensin-(1–7) enhances LTP in the hippocampus through the G-protein-coupled receptor Mas. Mol. Cell. Neurosci. 29, 427–435. 10.1016/j.mcn.2005.03.012 15950155

[B77] Heneka M. T. Carson M. J. El Khoury J. Landreth G. E. Brosseron F. Feinstein D. L.. (2015). Neuroinflammation in Alzheimer’s disease. Lancet Neurol. 14, 388–405. 10.1016/S1474-4422(15)70016-5 25792098 PMC5909703

[B78] Herrera M. Silva G. B. Garvin J. L. (2010). Angiotensin II stimulates thick ascending limb superoxide production via protein kinase C^α^-dependent NADPH oxidase activation. J. Biol. Chem. 285, 21323–21328. 10.1074/jbc.M110.109157 20448043 PMC2898432

[B79] Heymes C. Silvestre J. S. Llorens-Cortes C. Chevalier B. Marotte F. Levy B. I.. (1998). Cardiac senescence is associated with enhanced expression of angiotensin II receptor subtypes. Endocrinology 139, 2579–2587. 10.1210/en.139.5.2579 9564874

[B80] Hickman S. E. Kingery N. D. Ohsumi T. K. Borowsky M. L. Wang L. C. Means T. K.. (2013). The microglial sensome revealed by direct RNA sequencing. Nat. Neurosci. 16, 1896–1905. 10.1038/nn.3554 24162652 PMC3840123

[B81] Hoek R. M. Ruuls S. R. Murphy C. A. Wright G. J. Goddard R. Zurawski S. M.. (2000). Down-regulation of the macrophage lineage through interaction with OX2 (CD200). Science 290, 1768–1771. 10.1126/science.290.5497.1768 11099416

[B82] Hofman F. M. Hinton D. R. Johnson K. Merrill J. E. (1989). Tumor necrosis factor identified in multiple sclerosis brain. J. Exp. Med. 170, 607–612. 10.1084/jem.170.2.607 2754393 PMC2189402

[B83] Honing H. van den Berg T. K. van der Pol S. M. Dijkstra C. D. van der Kammen R. A. Collard J. G.. (2004). RhoA activation promotes transendothelial migration of monocytes via ROCK. J. Leukoc. Biol. 75, 523–528. 10.1189/jlb.0203054 14634067

[B84] Hoogwerf B. J. (2010). Renin-angiotensin system blockade and cardiovascular and renal protection. Am. J. Cardiol. 105, 30A–35A. 10.1016/j.amjcard.2009.10.009 20102971

[B85] Hu X. Liou A. K. Leak R. K. Xu M. An C. Suenaga J.. (2014). Neurobiology of microglial action in CNS injuries: receptor-mediated signaling mechanisms and functional roles. Prog. Neurobiol. 119–120, 60–84. 10.1016/j.pneurobio.2014.06.002 24923657 PMC4121732

[B86] Ishibashi K. Ishii K. Oda K. Kawasaki K. Mizusawa H. Ishiwata K. (2009). Regional analysis of age-related decline in dopamine transporters and dopamine D2-like receptors in human striatum. Synapse 63, 282–290. 10.1002/syn.20603 19116949

[B87] Iwanami J. Mogi M. Tsukuda K. Min L. J. Sakata A. Jing F.. (2010). Low dose of telmisartan prevents ischemic brain damage with peroxisome proliferator-activated receptor-γ activation in diabetic mice. J. Hypertens. 28, 1730–1737. 10.1097/hjh.0b013e32833a551a 20498620

[B88] Iwanami J. Mogi M. Tsukuda K. Wang X.-L. Nakaoka H. Kan-no H.. (2015). Direct angiotensin II type 2 receptor stimulation by compound 21 prevents vascular dementia. J. Am. Soc. Hypertens. 9, 250–256. 10.1016/j.jash.2015.01.010 25753301

[B89] Jiang C. Ting A. T. Seed B. (1998). PPAR-γ agonists inhibit production of monocyte inflammatory cytokines. Nature 391, 82–86. 10.1038/34184 9422509

[B90] Jin C. Z. Jang J. H. Wang Y. Kim J. G. Bae Y. M. Shi J.. (2012). Neuronal nitric oxide synthase is up-regulated by angiotensin II and attenuates NADPH oxidase activity and facilitates relaxation in murine left ventricular myocytes. J. Mol. Cell. Cardiol. 52, 1274–1281. 10.1016/j.yjmcc.2012.03.013 22484619

[B91] Joglar B. Rodriguez-Pallares J. Rodriguez-Perez A. I. Rey P. Guerra M. J. Labandeira-Garcia J. L. (2009). The inflammatory response in the MPTP model of Parkinson’s disease is mediated by brain angiotensin: relevance to progression of the disease. J. Neurochem. 109, 656–669. 10.1111/j.1471-4159.2009.05999.x 19245663

[B92] Jones E. S. Vinh A. McCarthy C. A. Gaspari T. A. Widdop R. E. (2008). AT2 receptors: functional relevance in cardiovascular disease. Pharmacol. Ther. 120, 292–316. 10.1016/j.pharmthera.2008.08.009 18804122 PMC7112668

[B93] Kehoe P. G. Wilcock G. K. (2007). Is inhibition of the renin-angiotensin system a new treatment option for Alzheimer’s disease? Lancet Neurol. 6, 373–378. 10.1016/s1474-4422(07)70077-7 17362841

[B94] Kerr D. S. Bevilaqua L. R. Bonini J. S. Rossato J. I. Köhler C. A. Medina J. H.. (2005). Angiotensin II blocks memory consolidation through an AT2 receptor-dependent mechanism. Psychopharmacology (Berl) 179, 529–535. 10.1007/s00213-004-2074-5 15551065

[B95] Kettenmann H. Hanisch U.-K. Noda M. Verkhratsky A. (2011). Physiology of microglia. Physiol. Rev. 91, 461–553. 10.1152/physrev.00011.2010 21527731

[B96] Kigerl K. A. Gensel J. C. Ankeny D. P. Alexander J. K. Donnelly D. J. Popovich P. G. (2009). Identification of two distinct macrophage subsets with divergent effects causing either neurotoxicity or regeneration in the injured mouse spinal cord. J Neurosci 29, 13435–13444. 10.1523/JNEUROSCI.3257-09.2009 19864556 PMC2788152

[B97] Kim J. Y. Kim N. Yenari M. A. (2015). Mechanisms and potential therapeutic applications of microglial activation after brain injury. CNS Neurosci. Ther. 21, 309–319. 10.1111/cns.12360 25475659 PMC4376565

[B98] Koenigsknecht-Talboo J. Landreth G. E. (2005). Microglial phagocytosis induced by fibrillar β-amyloid and IgGs are differentially regulated by proinflammatory cytokines. J. Neurosci. 25, 8240–8249. 10.1523/JNEUROSCI.1808-05.2005 16148231 PMC6725530

[B99] Kong J. Zhang K. Meng X. Zhang Y. Zhang C. (2015). Dose-dependent bidirectional effect of angiotensin IV on abdominal aortic aneurysm via variable angiotensin receptor stimulation. Hypertension 66, 617–626. 10.1161/HYPERTENSIONAHA.115.05482 26238445

[B100] Kostenis E. Milligan G. Christopoulos A. Sanchez-Ferrer C. F. Heringer-Walther S. Sexton P. M.. (2005). G-protein-coupled receptor Mas is a physiological antagonist of the angiotensin II type 1 receptor. Circulation 111, 1806–1813. 10.1161/01.cir.0000160867.23556.7d 15809376

[B101] Kubis N. Faucheux B. A. Ransmayr G. Damier P. Duyckaerts C. Henin D.. (2000). Preservation of midbrain catecholaminergic neurons in very old human subjects. Brain 123, 366–373. 10.1093/brain/123.2.366 10648443

[B102] Kumar A. Rassoli A. Raizada M. K. (1988). Angiotensinogen gene expression in neuronal and glial cells in primary cultures of rat brain. J. Neurosci. Res. 19, 287–290. 10.1002/jnr.490190302 3379645

[B103] Labandeira-García J. L. Garrido-Gil P. Rodriguez-Pallares J. Valenzuela R. Borrajo A. Rodríguez-Perez A. I. (2014). Brain renin-angiotensin system and dopaminergic cell vulnerability. Front. Neuroanat. 8:67. 10.3389/fnana.2014.00067 25071471 PMC4086395

[B104] Labandeira-Garcia J. L. Rodriguez-Pallares J. Dominguez-Meijide A. Valenzuela R. Villar-Cheda B. Rodriguez-Perez A. I. (2013). Dopamine-angiotensin interactions in the basal ganglia and their relevance for Parkinson’s disease. Mov. Disord. 28, 1337–1342. 10.1002/mds.25614 23925977

[B105] Labandeira-Garcia J. L. Rodriguez-Perez A. I. Valenzuela R. Costa-Besada M. A. Guerra M. J. (2016). Menopause and Parkinson’s disease. Interaction between estrogens and brain renin-angiotensin system in dopaminergic degeneration. Front. Neuroendocrinol. 43, 44–59. 10.1016/j.yfrne.2016.09.003 27693730

[B106] Labandeira-Garcia J. L. Rodríguez-Perez A. I. Villar-Cheda B. Borrajo A. Dominguez-Meijide A. Guerra M. J. (2015). Rho kinase and dopaminergic degeneration: a promising therapeutic target for Parkinson’s disease. Neuroscientist 21, 616–629. 10.1177/1073858414554954 25323761

[B107] Lawson L. J. Perry V. H. Dri P. Gordon S. (1990). Heterogeneity in the distribution and morphology of microglia in the normal adult mouse brain. Neuroscience 39, 151–170. 10.1016/0306-4522(90)90229-w 2089275

[B109] Liu M. Shi P. Sumners C. (2016). Direct anti-inflammatory effects of angiotensin-(1–7) on microglia. J. Neurochem. 136, 163–171. 10.1111/jnc.13386 26448556 PMC4688174

[B108] Liu J. Zhang P.-S. Yu Q. Liu L. Yang Y. Guo F.-M.. (2012). Losartan inhibits conventional dendritic cell maturation and Th1 and Th17 polarization responses: novel mechanisms of preventive effects on lipopolysaccharide-induced acute lung injury. Int. J. Mol. Med. 29, 269–276. 10.3892/ijmm.2011.818 22020765

[B110] Lopes K. O. Sparks D. L. Streit W. J. (2008). Microglial dystrophy in the aged and Alzheimer’s disease brain is associated with ferritin immunoreactivity. Glia 56, 1048–1060. 10.1002/glia.20678 18442088

[B111] Lopez-Real A. Rey P. Soto-Otero R. Mendez-Alvarez E. Labandeira-Garcia J. L. (2005). Angiotensin-converting enzyme inhibition reduces oxidative stress and protects dopaminergic neurons in a 6-hydroxydopamine rat model of Parkinsonism. J. Neurosci. Res. 81, 865–873. 10.1002/jnr.20598 16015598

[B112] Lu J. Wu L. Jiang T. Wang Y. Zhao H. Gao Q.. (2015). Angiotensin AT2 receptor stimulation inhibits activation of NADPH oxidase and ameliorates oxidative stress in rotenone model of Parkinson’s disease in CATH.a cells. Neurotoxicol. Teratol. 47, 16–24. 10.1016/j.ntt.2014.11.004 25446015

[B113] Ma L.-J. Corsa B. A. Zhou J. Yang H. Li H. Tang Y.-W.. (2011). Angiotensin type 1 receptor modulates macrophage polarization and renal injury in obesity. Am. J. Physiol. Renal Physiol. 300, F1203–F1213. 10.1152/ajprenal.00468.2010 21367915 PMC3094053

[B114] MacMicking J. Xie Q. W. Nathan C. (1997). Nitric oxide and macrophage function. Annu. Rev. Immunol. 15, 323–350. 10.1146/annurev.immunol.15.1.323 9143691

[B115] Mandrekar-Colucci S. Karlo J. C. Landreth G. E. (2012). Mechanisms underlying the rapid peroxisome proliferator-activated receptor-γ-mediated amyloid clearance and reversal of cognitive deficits in a murine model of Alzheimer’s disease. J. Neurosci. 32, 10117–10128. 10.1523/JNEUROSCI.5268-11.2012 22836247 PMC3433721

[B116] Mantovani A. Sica A. Sozzani S. Allavena P. Vecchi A. Locati M. (2004). The chemokine system in diverse forms of macrophage activation and polarization. Trends Immunol. 25, 677–686. 10.1016/j.it.2004.09.015 15530839

[B117] Maul B. Krause W. Pankow K. Becker M. Gembardt F. Alenina N.. (2005). Central angiotensin II controls alcohol consumption via its AT1 receptor. FASEB J. 19, 1474–1481. 10.1096/fj.05-3742com 16126915

[B118] McCormack A. L. Di Monte D. A. Delfani K. Irwin I. DeLanney L. E. Langston W. J.. (2004). Aging of the nigrostriatal system in the squirrel monkey. J. Comp. Neurol. 471, 387–395. 10.1002/cne.20036 15022260

[B119] McGuire S. O. Ling Z. D. Lipton J. W. Sortwell C. E. Collier T. J. Carvey P. M. (2001). Tumor necrosis factor α is toxic to embryonic mesencephalic dopamine neurons. Exp. Neurol. 169, 219–230. 10.1006/exnr.2001.7688 11358437

[B120] Mehlhase J. Gieche J. Widmer R. Grune T. (2006). Ferritin levels in microglia depend upon activation: modulation by reactive oxygen species. Biochim. Biophys. Acta 1763, 854–859. 10.1016/j.bbamcr.2006.04.012 16777245

[B121] Mehta P. K. Griendling K. K. (2007). Angiotensin II cell signaling: physiological and pathological effects in the cardiovascular system. Am. J. Physiol. Cell Physiol. 292, C82–C97. 10.1152/ajpcell.00287.2006 16870827

[B122] Mendelsohn F. A. Jenkins T. A. Berkovic S. F. (1993). Effects of angiotensin II on dopamine and serotonin turnover in the striatum of conscious rats. Brain Res. 613, 221–229. 10.1016/0006-8993(93)90902-y 7514480

[B123] Milsted A. Barna B. P. Ransohoff R. M. Brosnihan K. B. Ferrario C. M. (1990). Astrocyte cultures derived from human brain tissue express angiotensinogen mRNA. Proc. Natl. Acad. Sci. U S A 87, 5720–5723. 10.1073/pnas.87.15.5720 2377609 PMC54399

[B124] Min L. J. Mogi M. Iwai M. Horiuchi M. (2009). Signaling mechanisms of angiotensin II in regulating vascular senescence. Ageing Res. Rev. 8, 113–121. 10.1016/j.arr.2008.12.002 19162241

[B125] Mittelbronn M. Dietz K. Schluesener H. J. Meyermann R. (2001). Local distribution of microglia in the normal adult human central nervous system differs by up to one order of magnitude. Acta Neuropathol. 101, 249–255. 10.1007/s004010000284 11307625

[B126] Miyoshi M. Miyano K. Moriyama N. Taniguchi M. Watanabe T. (2008). Angiotensin type 1 receptor antagonist inhibits lipopolysaccharide-induced stimulation of rat microglial cells by suppressing nuclear factor κB and activator protein-1 activation. Eur. J. Neurosci. 27, 343–351. 10.1111/j.1460-9568.2007.06014.x 18190523

[B128] Mogi M. Harada M. Riederer P. Narabayashi H. Fujita K. Nagatsu T. (1994). Tumor necrosis factor-α (TNF-α) increases both in the brain and in the cerebrospinal fluid from parkinsonian patients. Neurosci. Lett. 165, 208–210. 10.1016/0304-3940(94)90746-3 8015728

[B127] Mogi M. Horiuchi M. (2009). Effects of angiotensin II receptor blockers on dementia. Hypertens. Res. 32, 738–740. 10.1038/hr.2009.110 19727113

[B129] Morale M. C. Serra P. A. L’Episcopo F. Tirolo C. Caniglia S. Testa N.. (2006). Estrogen, neuroinflammation and neuroprotection in Parkinson’s disease: glia dictates resistance versus vulnerability to neurodegeneration. Neuroscience 138, 869–878. 10.1016/j.neuroscience.2005.07.060 16337092

[B130] Mrak R. E. Landreth G. E. (2004). PPARγ, neuroinflammation and disease. J. Neuroinflammation 1:5. 10.1186/1742-2094-1-5 15285797 PMC483058

[B131] Muñoz A. Rey P. Guerra M. J. Mendez-Alvarez E. Soto-Otero R. Labandeira-Garcia J. L. (2006). Reduction of dopaminergic degeneration and oxidative stress by inhibition of angiotensin converting enzyme in a MPTP model of parkinsonism. Neuropharmacology 51, 112–120. 10.1016/j.neuropharm.2006.03.004 16678218

[B132] Münzel T. Keaney J. F. Jr. (2001). Are ACE inhibitors a “magic bullet” against oxidative stress? Circulation 104, 1571–1574. 10.1161/hc3801.095585 11571254

[B133] Nadjar A. Berton O. Guo S. Leneuve P. Dovero S. Diguet E.. (2009). IGF-1 signaling reduces neuro-inflammatory response and sensitivity of neurons to MPTP. Neurobiol. Aging 30, 2021–2030. 10.1016/j.neurobiolaging.2008.02.009 18394756

[B134] Nguyen G. (2011). Renin, (pro)renin and receptor: an update. Clin. Sci. (Lond) 120, 169–178. 10.1042/CS20100432 21087212

[B135] Nguyen G. Contrepas A. (2008). The (pro)renin receptors. J. Mol. Med. (Berl) 86, 643–646. 10.1007/s00109-008-0319-1 18322668

[B136] Nguyen G. Delarue F. Burcklé C. Bouzhir L. Giller T. Sraer J. D. (2002). Pivotal role of the renin/prorenin receptor in angiotensin II production and cellular responses to renin. J. Clin. Invest. 109, 1417–1427. 10.1172/jci0214276 12045255 PMC150992

[B137] Nimmerjahn A. Kirchhoff F. Helmchen F. (2005). Resting microglial cells are highly dynamic surveillants of brain parenchyma *in vivo*. Science 308, 1314–1318. 10.1126/science.1110647 15831717

[B138] Orihuela R. McPherson C. A. Harry G. J. (2016). Microglial M1/M2 polarization and metabolic states. Br. J. Pharmacol. 173, 649–665. 10.1111/bph.13139 25800044 PMC4742299

[B139] Oro C. Qian H. Thomas W. G. (2007). Type 1 angiotensin receptor pharmacology: signaling beyond G proteins. Pharmacol. Ther. 113, 210–226. 10.1016/j.pharmthera.2006.10.001 17125841 PMC7112676

[B140] Ouchi Y. Yoshikawa E. Sekine Y. Futatsubashi M. Kanno T. Ogusu T.. (2005). Microglial activation and dopamine terminal loss in early Parkinson’s disease. Ann. Neurol. 57, 168–175. 10.1002/ana.20338 15668962

[B141] Pang T. Benicky J. Wang J. Orecna M. Sanchez-Lemus E. Saavedra J. M. (2012). Telmisartan ameliorates lipopolysaccharide-induced innate immune response through peroxisome proliferator-activated receptor-γ activation in human monocytes. J. Hypertens. 30, 87–96. 10.1097/HJH.0b013e32834dde5f 22124178 PMC3237779

[B142] Peng J. Kimura B. Phillips M. I. (2002). The predominant role of brain angiotensinogen and angiotensin in environmentally induced hypertension. Regul. Pept. 110, 25–32. 10.1016/s0167-0115(02)00156-8 12468106

[B143] Phillips M. I. de Oliveira E. M. (2008). Brain renin angiotensin in disease. J. Mol. Med. (Berl) 86, 715–722. 10.1007/s00109-008-0331-5 18385968 PMC7095973

[B144] Platten M. Youssef S. Hur E. M. Ho P. P. Han M. H. Lanz T. V.. (2009). Blocking angiotensin-converting enzyme induces potent regulatory T cells and modulates TH1- and TH17-mediated autoimmunity. Proc. Natl. Acad. Sci. U S A 106, 14948–14953. 10.1073/pnas.0903958106 19706421 PMC2736463

[B145] Plumb R. D. El-Sherbeeny N. A. Dixon L. J. Hughes S. M. Devine A. B. Leahey W. J.. (2005). NAD(P)H-dependent superoxide production in platelets: the role of angiotensin II and protein kinase C. Clin. Biochem. 38, 607–613. 10.1016/j.clinbiochem.2005.04.012 15922319

[B146] Porrini V. Lanzillotta A. Branca C. Benarese M. Parrella E. Lorenzini L.. (2015). CHF5074 (CSP-1103) induces microglia alternative activation in plaque-free Tg2576 mice and primary glial cultures exposed to β-amyloid. Neuroscience 302, 112–120. 10.1016/j.neuroscience.2014.10.029 25450955

[B147] Prinz M. Priller J. (2014). Microglia and brain macrophages in the molecular age: from origin to neuropsychiatric disease. Nat. Rev. Neurosci. 15, 300–312. 10.1038/nrn3722 24713688

[B148] Puche J. E. García-Fernández M. Muntané J. Rioja J. González-Barón S. Castilla Cortazar I. (2008). Low doses of insulin-like growth factor-I induce mitochondrial protection in aging rats. Endocrinology 149, 2620–2627. 10.3748/wjg.14.2731 18276748

[B149] Qin L. Liu Y. Wang T. Wei S. J. Block M. L. Wilson B.. (2004). NADPH oxidase mediates lipopolysaccharide-induced neurotoxicity and proinflammatory gene expression in activated microglia. J. Biol. Chem. 279, 1415–1421. 10.1074/jbc.M307657200 14578353

[B150] Qin L. Wu X. Block M. L. Liu Y. Breese G. R. Hong J. S.. (2007). Systemic LPS causes chronic neuroinflammation and progressive neurodegeneration. Glia 55, 453–462. 10.1002/glia.20467 17203472 PMC2871685

[B151] Quesada A. Romeo H. E. Micevych P. (2007). Distribution and localization patterns of estrogen receptor-β and insulin-like growth factor-1 receptors in neurons and glial cells of the female rat substantia nigra: localization of ERβ and IGF-1R in substantia nigra. J. Comp. Neurol. 503, 198–208. 10.1002/cne.21358 17480015 PMC2907103

[B152] Rajaram M. V. Brooks M. N. Morris J. D. Torrelles J. B. Azad A. K. Schlesinger L. S. (2010). Mycobacterium tuberculosis activates human macrophage peroxisome proliferator-activated receptor γ linking mannose receptor recognition to regulation of immune responses. J. Immunol. 185, 929–942. 10.4049/jimmunol.1000866 20554962 PMC3014549

[B153] Ransohoff R. M. Brown M. A. (2012). Innate immunity in the central nervous system. J. Clin. Invest. 122, 1164–1171. 10.1172/JCI58644 22466658 PMC3314450

[B154] Ransohoff R. M. Perry V. H. (2009). Microglial physiology: unique stimuli, specialized responses. Annu. Rev. Immunol. 27, 119–145. 10.1146/annurev.immunol.021908.132528 19302036

[B155] Re R. N. (2004). Tissue renin angiotensin systems. Med. Clin. North Am. 88, 19–38. 10.1016/S0025-7125(03)00124-X 14871049

[B156] Reeves E. P. Lu H. Jacobs H. L. Messina C. G. Bolsover S. Gabella G.. (2002). Killing activity of neutrophils is mediated through activation of proteases by K^+^ flux. Nature 416, 291–297. 10.1038/416291a 11907569

[B157] Rey P. Lopez-Real A. Sanchez-Iglesias S. Muñoz A. Soto-Otero R. Labandeira-Garcia J. L. (2007). Angiotensin type-1-receptor antagonists reduce 6-hydroxydopamine toxicity for dopaminergic neurons. Neurobiol. Aging 28, 555–567. 10.1016/j.neurobiolaging.2006.02.018 16621167

[B158] Ricote M. Li A. C. Willson T. M. Kelly C. J. Glass C. K. (1998). The peroxisome proliferator-activated receptor-γ is a negative regulator of macrophage activation. Nature 391, 79–82. 10.1038/34178 9422508

[B159] Rietdijk C. D. Van Wezel R. J. A. Garssen J. Kaneveld A. D. (2016). Neuronal toll-like receptors and neuro-immunity in Parkinson’s disease, Alzheimer’s disease and stroke. Neuroimmunol. Neuroinflammation 3, 27–37. 10.20517/2347-8659.2015.28

[B160] Rodriguez-Pallares J. Parga J. A. Joglar B. Guerra M. J. Labandeira-Garcia J. L. (2012). Mitochondrial ATP-sensitive potassium channels enhance angiotensin-induced oxidative damage and dopaminergic neuron degeneration. Relevance for aging-associated susceptibility to Parkinson’s disease. Age (Dordr) 34, 863–880. 10.1007/s11357-011-9284-7 21713375 PMC3682060

[B161] Rodriguez-Pallares J. Parga J. A. Muñoz A. Rey P. Guerra M. J. Labandeira-Garcia J. L. (2007). Mechanism of 6-hydroxydopamine neurotoxicity: the role of NADPH oxidase and microglial activation in 6-hydroxydopamine-induced degeneration of dopaminergic neurons. J. Neurochem. 103, 145–156. 10.1111/j.1471-4159.2007.04699.x 17573824

[B162] Rodriguez-Pallares J. Quiroz C. R. Parga J. A. Guerra M. J. Labandeira-Garcia J. L. (2004). Angiotensin II increases differentiation of dopaminergic neurons from mesencephalic precursors via angiotensin type 2 receptors. Eur. J. Neurosci. 20, 1489–1498. 10.1111/j.1460-9568.2004.03621.x 15355316

[B163] Rodriguez-Pallares J. Rey P. Parga J. A. Muñoz A. Guerra M. J. Labandeira-Garcia J. L. (2008). Brain angiotensin enhances dopaminergic cell death via microglial activation and NADPH-derived ROS. Neurobiol. Dis. 31, 58–73. 10.1016/j.nbd.2008.03.003 18499466

[B164] Rodriguez-Perez A. I. Borrajo A. Diaz-Ruiz C. Garrido-Gil P. Labandeira-Garcia J. L. (2016). Crosstalk between insulin-like growth factor-1 and angiotensin-II in dopaminergic neurons and glial cells: role in neuroinflammation and aging. Oncotarget 7, 30049–30067. 10.18632/oncotarget.9174 27167199 PMC5058663

[B165] Rodriguez-Perez A. I. Borrajo A. Rodriguez-Pallares J. Guerra M. J. Labandeira-Garcia J. L. (2015a). Interaction between NADPH-oxidase and Rho-kinase in angiotensin II-induced microglial activation. Glia 63, 466–482. 10.1002/glia.22765 25377425

[B166] Rodriguez-Perez A. I. Borrajo A. Valenzuela R. Lanciego J. L. Labandeira-Garcia J. L. (2015b). Critical period for dopaminergic neuroprotection by hormonal replacement in menopausal rats. Neurobiol. Aging 36, 1194–1208. 10.1016/j.neurobiolaging.2014.10.028 25432430

[B167] Rodriguez-Perez A. I. Dominguez-Meijide A. Lanciego J. L. Guerra M. J. Labandeira-Garcia J. L. (2013). Inhibition of Rho kinase mediates the neuroprotective effects of estrogen in the MPTP model of Parkinson’s disease. Neurobiol. Dis. 58, 209–219. 10.1016/j.nbd.2013.06.004 23774254

[B168] Rodriguez-Perez A. I. Valenzuela R. Villar-Cheda B. Guerra M. J. Labandeira-Garcia J. L. (2012). Dopaminergic neuroprotection of hormonal replacement therapy in young and aged menopausal rats: role of the brain angiotensin system. Brain 135, 124–138. 10.1093/brain/awr320 22189567

[B169] Rodriguez-Perez A. I. Valenzuela R. Villar-Cheda B. Guerra M. J. Lanciego J. L. Labandeira-Garcia J. L. (2010). Estrogen and angiotensin interaction in the substantia nigra. Relevance to postmenopausal Parkinson’s disease. Exp. Neurol. 224, 517–526. 10.1016/j.expneurol.2010.05.015 20580712

[B170] Rompe F. Artuc M. Hallberg A. Alterman M. Ströder K. Thöne-Reineke C.. (2010). Direct angiotensin II type 2 receptor stimulation acts anti-inflammatory through epoxyeicosatrienoic acid and inhibition of nuclear factor kappaB. Hypertension 55, 924–931. 10.1161/HYPERTENSIONAHA.109.147843 20157051

[B171] Ruiz-Ortega M. Lorenzo O. Suzuki Y. Rupérez M. Egido J. (2001). Proinflammatory actions of angiotensins. Curr. Opin. Nephrol. Hypertens. 10, 321–329. 10.1097/00041552-200105000-00005 11342793

[B172] Saab Y. B. Gard P. R. Yeoman M. S. Mfarrej B. El-Moalem H. Ingram M. J. (2007). Renin-angiotensin-system gene polymorphisms and depression. Prog. Neuropsychopharmacol. Biol. Psychiatry 31, 1113–1118. 10.1016/j.pnpbp.2007.04.002 17499413

[B173] Saavedra J. M. (2012). Angiotensin II AT_1_ receptor blockers as treatments for inflammatory brain disorders. Clin. Sci. 123, 567–590. 10.1042/CS20120078 22827472 PMC3501743

[B174] Sánchez-Iglesias S. Rey P. Méndez-Alvarez E. Labandeira-García J. L. Soto-Otero R. (2007). Time-course of brain oxidative damage caused by intrastriatal administration of 6-hydroxydopamine in a rat model of Parkinson’s disease. Neurochem. Res. 32, 99–105. 10.1007/s11064-006-9232-6 17160721

[B175] Santos R. A. Simoes e Silva A. C. Maric C. Silva D. M. Machado R. P. de Buhr I.. (2003). Angiotensin-(1–7) is an endogenous ligand for the G protein-coupled receptor Mas. Proc. Natl. Acad. Sci. U S A 100, 8258–8263. 10.1073/pnas.1432869100 12829792 PMC166216

[B176] Savaskan E. (2005). The role of the brain renin-angiotensin system in neurodegenerative disorders. Curr. Alzheimer Res. 2, 29–35. 10.2174/1567205052772740 15977987

[B177] Schintu N. Frau L. Ibba M. Caboni P. Garau A. Carboni E.. (2009). PPAR-γ-mediated neuroprotection in a chronic mouse model of Parkinson’s disease. Eur. J. Neurosci. 29, 954–963. 10.1111/j.1460-9568.2009.06657.x 19245367

[B178] Seshiah P. N. Weber D. S. Rocic P. Valppu L. Taniyama Y. Griendling K. K. (2002). Angiotensin II stimulation of NAD(P)H oxidase activity: upstream mediators. Circ. Res. 91, 406–413. 10.1161/01.res.0000033523.08033.16 12215489

[B179] Shan Z. Cuadra A. E. Sumners C. Raizada M. K. (2008). Characterization of a functional (pro)renin receptor in rat brain neurons. Exp. Physiol. 93, 701–708. 10.1113/expphysiol.2008.041988 18326551 PMC3130537

[B180] Sheh Y. L. Hsu C. Chan S. H. Chan J. Y. (2007). NADPH oxidase- and mitochondrion-derived superoxide at rostral ventrolateral medulla in endotoxin-induced cardiovascular depression. Free Radic. Biol. Med. 42, 1610–1623. 10.1016/j.freeradbiomed.2007.02.019 17448908

[B181] Shi P. Grobe J. L. Desland F. A. Zhou G. Shen X. Z. Shan Z.. (2014). Direct pro-inflammatory effects of prorenin on microglia. PLoS One 9:e92937. 10.1371/journal.pone.0092937 25302502 PMC4193744

[B182] Shrestha S. Noh J. M. Kim S. Y. Ham H. Y. Kim Y. J. Yun Y. J.. (2016). Angiotensin converting enzyme inhibitors and angiotensin II receptor antagonist attenuate tumor growth via polarization of neutrophils toward an antitumor phenotype. Oncoimmunology 5:e1067744. 10.1080/2162402x.2015.1067744 26942086 PMC4760329

[B183] Sica A. Bronte V. (2007). Altered macrophage differentiation and immune dysfunction in tumor development. J. Clin. Invest. 117, 1155–1166. 10.1172/JCI31422 17476345 PMC1857267

[B184] Sica A. Mantovani A. (2012). Macrophage plasticity and polarization: *in vivo* veritas. J. Clin. Invest. 122, 787–795. 10.1172/JCI59643 22378047 PMC3287223

[B185] Sohn H. Y. Raff U. Hoffmann A. Gloe T. Heermeier K. Galle J.. (2000). Differential role of angiotensin II receptor subtypes on endothelial superoxide formation. Br. J. Pharmacol. 131, 667–672. 10.1038/sj.bjp.0703566 11030714 PMC1572372

[B186] Sonsalla P. K. Coleman C. Wong L. Y. Harris S. L. Richardson J. R. Gadad B. S.. (2013). The angiotensin converting enzyme inhibitor captopril protects nigrostriatal dopamine neurons in animal models of parkinsonism. Exp. Neurol. 250, 376–383. 10.1016/j.expneurol.2013.10.014 24184050 PMC3889207

[B187] Stegbauer J. Lee D. H. Seubert S. Ellrichmann G. Manzel A. Kvakan H.. (2009). Role of the renin-angiotensin system in autoimmune inflammation of the central nervous system. Proc. Natl. Acad. Sci. U S A 106, 14942–14947. 10.1073/pnas.0903602106 19706425 PMC2736426

[B188] Stornetta R. L. Hawelu-Johnson C. L. Guyenet P. G. Lynch K. R. (1988). Astrocytes synthesize angiotensinogen in brain. Science 242, 1444–1446. 10.1126/science.3201232 3201232

[B189] Sugama S. Yang L. Cho B. P. DeGiorgio L. A. Lorenzl S. Albers D. S.. (2003). Age-related microglial activation in 1-methyl-4-phenyl-1,2,3,6-tetrahydropyridine (MPTP)-induced dopaminergic neurodegeneration in C57BL/6 mice. Brain Res. 964, 288–294. 10.1016/s0006-8993(02)04085-4 12576189

[B191] Suh Y. Atzmon G. Cho M. O. Hwang D. Liu B. Leahy D. J.. (2008). Functionally significant insulin-like growth factor I receptor mutations in centenarians. Proc. Natl. Acad. Sci. U S A 105, 3438–3442. 10.1073/pnas.0705467105 18316725 PMC2265137

[B190] Suh H. S. Zhao M. L. Derico L. Choi N. Lee S. C. (2013). Insulin-like growth factor 1 and 2 (IGF1, IGF2) expression in human microglia: differential regulation by inflammatory mediators. J. Neuroinflammation 10:37. 10.1186/1742-2094-10-37 23497056 PMC3607995

[B192] Suzuki S. Brown C. M. Dela Cruz C. D. Yang E. Bridwell D. A. Wise P. M. (2007). Timing of estrogen therapy after ovariectomy dictates the efficacy of its neuroprotective and antiinflammatory actions. Proc. Natl. Acad. Sci. U S A 104, 6013–6018. 10.1073/pnas.0610394104 17389368 PMC1851608

[B193] Suzuki Y. Ruiz-Ortega M. Lorenzo O. Ruperez M. Esteban V. Egido J. (2003). Inflammation and angiotensin II. Int. J. Biochem. Cell Biol. 35, 881–900. 10.1016/S1357-2725(02)00271-6 12676174

[B194] Tang Y. Le W. (2016). Differential roles of M1 and M2 microglia in neurodegenerative diseases. Mol. Neurobiol. 53, 1181–1194. 10.1007/s12035-014-9070-5 25598354

[B195] Tannahill G. M. Iraci N. Gaude E. Frezza C. Pluchino S. (2015). Metabolic reprograming of mononuclear phagocytes in progressive multiple sclerosis. Front. Immunol. 6:106. 10.3389/fimmu.2015.00106 25814990 PMC4356156

[B197] Tao Y. Pinzi V. Bourhis J. Deutsch E. (2007). Mechanisms of disease: signaling of the insulin-like growth factor 1 receptor pathway—therapeutic perspectives in cancer. Nat. Clin. Pract. Oncol. 4, 591–602. 10.1038/ncponc0934 17898809

[B196] Tao L. Qiu Y. Fu X. Lin R. Lei C. Wang J.. (2016). Angiotensin-converting enzyme 2 activator diminazene aceturate prevents lipopolysaccharide-induced inflammation by inhibiting MAPK and NF-κB pathways in human retinal pigment epithelium. J. Neuroinflammation 13:35. 10.1186/s12974-016-0489-7 26862037 PMC4748536

[B198] Thomas W. G. Greenland K. J. Shinkel T. A. Sernia C. (1992). Angiotensinogen is secreted by pure rat neuronal cell cultures. Brain Res. 588, 191–200. 10.1016/0006-8993(92)91575-y 1393575

[B199] Thompson M. M. Oyama T. T. Kelly F. J. Kennefick T. M. Anderson S. (2000). Activity and responsiveness of the renin-angiotensin system in the aging rat. Am. J. Physiol. Regul. Integr. Comp. Physiol. 279, R1787–R1794. 10.1007/978-1-60761-186-8_16 11049862

[B200] Torika N. Asraf K. Roasso E. Danon A. Fleisher-Berkovich S. (2016). Angiotensin converting enzyme inhibitors ameliorate brain inflammation associated with microglial activation: possible implications for Alzheimer’s disease. J. Neuroimmune Pharmacol. 11, 774–785. 10.1007/s11481-016-9703-8 27562846

[B201] Touyz R. M. (2004). Reactive oxygen species and angiotensin II signaling in vascular cells—implications in cardiovascular disease. Braz. J. Med. Biol. Res. 37, 1263–1273. 10.1590/s0100-879x2004000800018 15273829

[B202] Tran D. Bergholz J. Zhang H. He H. Wang Y. Zhang Y.. (2014). Insulin-like growth factor-1 regulates the SIRT1–p53 pathway in cellular senescence. Aging Cell 13, 669–678. 10.1111/acel.12219 25070626 PMC4118446

[B203] Umemoto S. (2008). Angiotensin II type 1 (AT1) receptor deficiency halts the progression of age-related atherosclerosis in hypercholesterolemia: molecular link between the AT1 receptor and hypercholesterolemia. Hypertens. Res. 31, 1495–1497. 10.1291/hypres.31.1495 18971522

[B204] Unger T. Chung O. Csikos T. Culman J. Gallinat S. Gohlke P.. (1996). Angiotensin receptors. J. Hypertens. Suppl. 14, S95–S103. 9120691

[B205] Valenzuela R. Barroso-Chinea P. Villar-Cheda B. Joglar B. Muñoz A. Lanciego J. L.. (2010). Location of prorenin receptors in primate substantia nigra: effects on dopaminergic cell death. J. Neuropathol. Exp. Neurol. 69, 1130–1142. 10.1097/NEN.0b013e3181fa0308 20940627

[B206] Valenzuela R. Costa-Besada M. A. Iglesias-Gonzalez J. Perez-Costas E. Villar-Cheda B. Garrido-Gil P.. (2016). Mitochondrial angiotensin receptors in dopaminergic neurons. Role in cell protection and aging-related vulnerability to neurodegeneration. Cell Death Dis. 7:e2427. 10.1038/cddis.2016.327 27763643 PMC5133991

[B207] Vegeto E. Benedusi V. Maggi A. (2008). Estrogen anti-inflammatory activity in brain: a therapeutic opportunity for menopause and neurodegenerative diseases. Front. Neuroendocrinol. 29, 507–519. 10.1016/j.yfrne.2008.04.001 18522863 PMC2630539

[B208] Villapol S. Saavedra J. M. (2015). Neuroprotective effects of angiotensin receptor blockers. Am. J. Hypertens. 28, 289–299. 10.1093/ajh/hpu197 25362113

[B209] Villar-Cheda B. Dominguez-Meijide A. Joglar B. Rodriguez-Perez A. I. Guerra M. J. Labandeira-Garcia J. L. (2012a). Involvement of microglial RhoA/Rho-kinase pathway activation in the dopaminergic neuron death. Role of angiotensin via angiotensin type 1 receptors. Neurobiol. Dis. 47, 268–279. 10.1016/j.nbd.2012.04.010 22542954

[B213] Villar-Cheda B. Valenzuela R. Rodriguez-Perez A. I. Guerra M. J. Labandeira-Garcia J. L. (2012b). Aging-related changes in the nigral angiotensin system enhances proinflammatory and pro-oxidative markers and 6-OHDA-induced dopaminergic degeneration. Neurobiol. Aging 33, 204.e1–204.e11. 10.1016/j.neurobiolaging.2010.08.006 20888078

[B210] Villar-Cheda B. Dominguez-Meijide A. Valenzuela R. Granado N. Moratalla R. Labandeira-Garcia J. L. (2014). Aging-related dysregulation of dopamine and angiotensin receptor interaction. Neurobiol. Aging 35, 1726–1738. 10.1016/j.neurobiolaging.2014.01.017 24529758

[B211] Villar-Cheda B. Rodríguez-Pallares J. Valenzuela R. Muñoz A. Guerra M. J. Baltatu O. C.. (2010). Nigral and striatal regulation of angiotensin receptor expression by dopamine and angiotensin in rodents: implications for progression of Parkinson’s disease. Eur. J. Neurosci. 32, 1695–1706. 10.1111/j.1460-9568.2010.07448.x 20964730

[B212] Villar-Cheda B. Sousa-Ribeiro D. Rodriguez-Pallares J. Rodriguez-Perez A. I. Guerra M. J. Labandeira-Garcia J. L. (2009). Aging and sedentarism decrease vascularization and VEGF levels in the rat substantia nigra. Implications for Parkinson’s disease. J. Cereb. Blood Flow Metab. 29, 230–234. 10.1038/jcbfm.2008.127 18957989

[B214] Villela D. Leonhardt J. Patel N. Joseph J. Kirsch S. Hallberg A.. (2015). Angiotensin type 2 receptor (AT2R) and receptor Mas: a complex liaison. Clin. Sci. (Lond) 128, 227–234. 10.1042/CS20130515 25328009

[B215] Vinciguerra M. Santini M. P. Claycomb W. C. Ladurner A. G. Rosenthal N. (2009). Local IGF-1 isoform protects cardiomyocytes from hypertrophic and oxidative stresses via SirT1 activity. Aging (Albany NY) 2, 43–62. 10.18632/aging.100107 20228935 PMC2837204

[B216] Von Bohlen und Halbach O. Walther T. Bader M. Albrecht D. (2000). Interaction between *Mas* and the angiotensin AT1 receptor in the amygdala. J. Neurophysiol. 83, 2012–2021. 10758111 10.1152/jn.2000.83.4.2012

[B217] Vowinckel E. Reutens D. Becher B. Verge G. Evans A. Owens T.. (1997). PK11195 binding to the peripheral benzodiazepine receptor as a marker of microglia activation in multiple sclerosis and experimental autoimmune encephalomyelitis. J. Neurosci. Res. 50, 345–353. 10.1002/(SICI)1097-4547(19971015)50:2<345::AID-JNR22>3.3.CO;2-S 9373043

[B219] Wang Y. Chan G. L. Holden J. E. Dobko T. Mak E. Schulzer M.. (1998). Age-dependent decline of dopamine D1 receptors in human brain: a PET study. Synapse 30, 56–61. 10.1002/(SICI)1098-2396(199809)30:1<56::AID-SYN7>3.0.CO;2-J 9704881

[B218] Wang J. Pang T. Hafko R. Benicky J. Sanchez-Lemus E. Saavedra J. M. (2014). Telmisartan ameliorates glutamate-induced neurotoxicity: roles of AT(1) receptor blockade and PPARγ activation. Neuropharmacology 79, 249–261. 10.1016/j.neuropharm.2013.11.022 24316465 PMC3950310

[B220] West A. P. Brodsky I. E. Rahner C. Woo D. K. Erdjument-Bromage H. Tempst P.. (2011). TLR signalling augments macrophage bactericidal activity through mitochondrial ROS. Nature 472, 476–480. 10.1038/nature09973 21525932 PMC3460538

[B221] Wright J. W. Kawas L. H. Harding J. W. (2015). The development of small molecule angiotensin IV analogs to treat Alzheimer’s and Parkinson’s diseases. Prog. Neurobiol. 125, 26–46. 10.1016/j.pneurobio.2014.11.004 25455861

[B223] Wu L. Iwai M. Li Z. Shiuchi T. Min L. J. Cui T. X.. (2004). Regulation of inhibitory protein-kappaB and monocyte chemoattractant protein-1 by angiotensin II type 2 receptor-activated Src homology protein tyrosine phosphatase-1 in fetal vascular smooth muscle cells. Mol. Endocrinol. 18, 666–678. 10.1210/me.2003-0053 14684844

[B222] Wu D. C. Teismann P. Tieu K. Vila M. Jackson-Lewis V. Ischiropoulos H.. (2003). NADPH oxidase mediates oxidative stress in the 1-methyl-4-phenyl-1,2,3,6-tetrahydropyridine model of Parkinson’s disease. Proc. Natl. Acad. Sci. U S A 100, 6145–6150. 10.1073/pnas.0937239100 12721370 PMC156340

[B224] Xu Y. Xu Y. Wang Y. Wang Y. He L. Jiang Z.. (2015). Telmisartan prevention of LPS-induced microglia activation involves M2 microglia polarization via CaMKKβ-dependent AMPK activation. Brain Behav. Immun. 50, 298–313. 10.1016/j.bbi.2015.07.015 26188187

[B225] Yan J. Zhou X. Guo J. J. Mao L. Wang Y. J. Sun J.. (2012). Nogo-66 inhibits adhesion and migration of microglia via GTPase Rho pathway *in vitro*. J. Neurochem. 120, 721–731. 10.1111/j.1471-4159.2011.07619.x 22145612

[B226] Yang J. Chen C. Ren H. Han Y. He D. Zhou L.. (2012). Angiotensin II AT(2) receptor decreases AT_1_ receptor expression and function via nitric oxide/cGMP/Sp1 in renal proximal tubule cells from Wistar-Kyoto rats. J. Hypertens. 30, 1176–1184. 10.1097/HJH.0b013e3283532099 22504846 PMC3705562

[B227] Zalba G. San José G. Moreno M. U. Fortuño M. A. Fortuño A. Beaumont F. J.. (2001). Oxidative stress in arterial hypertension: role of NAD(P)H oxidase. Hypertension 38, 1395–1399. 10.1161/hy1201.099611 11751724

[B228] Zawada W. M. Banninger G. P. Thornton J. Marriott B. Cantu D. Rachubinski A. L.. (2011). Generation of reactive oxygen species in 1-methyl-4-phenylpyridinium (MPP+) treated dopaminergic neurons occurs as an NADPH oxidase-dependent two-wave cascade. J. Neuroinflammation 8:129. 10.1186/1742-2094-8-129 21975039 PMC3198931

[B229] Zecca L. Gallorini M. Schünemann V. Trautwein A. X. Gerlach M. Riederer P.. (2001). Iron, neuromelanin and ferritin content in the substantia nigra of normal subjects at different ages: consequences for iron storage and neurodegenerative processes. J. Neurochem. 76, 1766–1773. 10.1046/j.1471-4159.2001.00186.x 11259494

[B230] Zhu T. Miller A. G. Deliyanti D. Berka D. R. Agrotis A. Campbell D. J.. (2015). Prorenin stimulates a pro-angiogenic and pro-inflammatory response in retinal endothelial cells and an M1 phenotype in retinal microglia. Clin. Exp. Pharmacol. Physiol. 42, 537–548. 10.1111/1440-1681.12376 25707593

